# Development and evaluation of machine learning models for individualized prediction of myopia control efficacy treated with overnight orthokeratology

**DOI:** 10.3389/fmed.2025.1559435

**Published:** 2025-05-12

**Authors:** Lan Zhang, Mingjun Gao, Yiru Wang, Siqi Zhang, Huailin Zhu, Qi Zhao

**Affiliations:** Department of Ophthalmology, The Second Hospital of Dalian Medical University, Dalian, China

**Keywords:** machine learning, myopia, orthokeratology, axial length, high-order aberration

## Abstract

**Purpose:**

The primary objective of this study is to develop a predictive model utilizing fundamental clinical and ocular measurements to predict the effect of overnight orthokeratology on myopia control. Accordingly, this study aims to assist ophthalmologists in selecting adolescent myopia control methods.

**Methods:**

This retrospective study used one-year follow-up data of 225 myopia children treated with orthokeratology. Using the random sampling method, 225 samples were randomly divided into a training set (*n* = 180) and a test set (*n* = 45). LASSO regression identified predictive factors correlated with controlling myopia. The final features are input into the machine learning model for prediction model construction to predict 1-year axial length elongation. The prediction performance was evaluated according to the accuracy and AUC of the training set and the test set. DCA was used to assess the clinical benefits of the model.

**Results:**

Five features (age, diopter, flat keratometry, corneal higher-order aberrations (6 mm), and intraocular trefoil (6 mm) were used to build the machine learning model (*p* < 0.01)). Based on the accuracy, ROC, and DCA curves, the prediction performance and clinical practicability of five prediction models: KNN, SVM, RF, Extra Trees, and XGBoost were compared. In the DCA, all machine learning models consistently achieved greater net benefits within the clinical threshold range. SVM demonstrated the highest predictive quality with an AUC of 0.877 in the training and 0.828 in the external validation set.

**Conclusion:**

We developed and validated several prediction models for individualized prediction of myopia control efficacy treated with overnight orthokeratology through machine learning, using easily obtained clinical and corneal topography features. This cost effective strategy helps ophthalmologists predict the effect of using orthokeratology in children, and make timely adjustments to myopia control methods. The differential features selected by this model can also provide insights for optimizing lens design.

## Introduction

In recent years, the prevalence of myopia in adolescents has been on the rise, especially in urbanized areas of Asia. In these areas, the myopia prevalence in young people is about 80 -90%, and the prevalence of high myopia is higher (10–20%) (1). Unfortunately, patients with high myopia have an increased risk of complications that can lead to irreversible blindness, such as glaucoma, myopic macular degeneration, and high myopia-related optic neuropathy (2). Therefore, it is very important to implement strategies to reduce the progression of low myopia to high myopia in adolescents, because low myopia is the main modifiable risk factor for pathological myopia (3).

One strategy is orthokeratology (ortho-k), which involves wearing a reverse geometric lens made of a rigid material with high oxygen permeability overnight to reshape the anterior surface of the cornea. This process effectively flattens the central cornea when the lens is removed in the morning, providing a temporary correction of refractive error (4). Recent randomized clinical trials have confirmed the validity of orthokeratology in controlling the progression of myopia in children (5). Orthokeratology has the potential to slow down the progression of myopia in children and adolescents, and the effect at an early age (6–8 years old) is more significant (6).

However, the exact mechanism by which ortho-k slows the progression of myopia remains unclear. Studies in primates have shown that visual signals from the periphery can override visual signals from the central retina and alter central refractive development (7). This leads to the hypothesis that peripheral treatment strategies are effective in slowing the progression of myopia (8). However, the role of the peripheral retina in regulating eye growth during ortho-k is still controversial. Longitudinal studies have shown that relative hyperopia defocus has nothing to do with the onset of myopia progression in children (9). In addition, recent studies have shown that higher-order aberrations may affect axial length growth and the therapeutic effect of myopia control interventions (10). Clinical studies have provided evidence to support the effectiveness of peripheral treatment strategies in slowing the progression of myopia.

As a matter of fact, during ortho-k treatment, there are significant differences in the control effect of myopia among different individuals. Clinical studies have shown that compared with the matched group with monovision glasses, the myopia progression of the ortho-k group was reduced by 33 -63% (11). This is mainly due to the lack of individualized design in ortho-k because the parameters affecting the mitigation of axial elongation are still poorly understood. To solve this problem, a series of predictors have been proposed to explain the individual differences in the efficacy of ortho-k including age, baseline axial length, central corneal thickness (12), baseline pupil area, zone-3 mm flat K (13) and corneal power change at different area (14).

It is well known that predictive models are effective tools for clinical risk assessment, decision selection, and benefit evaluation. In the medical and health system, it can play a guiding role in disease prevention, screening, diagnosis, severity, treatment efficacy, and prognosis (15). The purpose of machine learning is to improve the performance of the machine by using experience and existing large amounts of data. It can learn actively according to sample information and improve the accuracy of decision-making (16). At present, scholars have studied the prediction model for the therapeutic effect of orthokeratology, but the data collection is not comprehensive enough.

Based on the analysis of the above research status, to accurately predict the efficacy of orthokeratology, we developed and validated predictive models to accurately predict the myopia control effect of ortho-k by collecting corneal topography and demographic information collected at the first visit before treatment. In terms of clinical significance, the standardized model can provide a reference for ophthalmologists to choose the method of controlling myopia. On the other hand, the influence variables demonstrated by the models can provide valuable insights for lens designers to optimize lens design.

## Materials and methods

### Patient

This retrospective study was approved by the Ethics Committee of the Second Hospital of Dalian Medical University, following the principle of Helsinki Declaration and participants less than 8 years old we obtained the informed consent of the legal guardian of the participants; informed consent was obtained from participants aged 8 years and above and their legal guardians. The data of this study were derived from the institutional ophthalmology database, covering medical records from 2020 to 2023. A total of 225 subjects who underwent comprehensive ophthalmic examination and met the inclusion criteria were included in the study. Inclusion criteria: (1) spherical equivalent (SER) -1.00 ~ −6.00D; (2) astigmatism < 1.75D; (3) anisometropia ≤ 1.50D; (4) corrected distant vision ≥ 1.0; (5) individuals aged from 7 to 12 years old; (6) have finished 1-year visit. The exclusion criteria were as follows: (1) a previous history of ortho-k lens or soft contact lens wear, (2) discontinuation of ortho-k lens use during study period; (3) prescription modification beyond 1 month of ortho-k lens wear; (4) in addition to myopia, there were other eye or systemic diseases or ophthalmic operation; (5) use of anticholinergic or cholinergic drugs, such as atropine, pilocarpine, etc., within the past 3 months that interfere with the control effect.

### Group

This study was grouped according to the axial length. The axial length is an index recognized at home and abroad that is positively correlated with the progression of myopia (29). Baseline axial length (AL) and 1-year AL data were collected using a biometric instrument (LS 900). Each visit was continuously measured 5 times, and the average value was taken as the representative value. After 1 year of treatment, the axial/axial ratio before treatment was ranked from high to low, and the median value was divided into two groups: good effect and poor effect.

### Lens

The orthokeratology lens selected in this study was Paragon CRT with HDS100 paflufocon D lens material, with the oxygen transmission coefficient of 100, the light transmittance of 95%, the refractive index of 1.442, wetting angle of 42°, a ratio of 1.1, average total lens diameter of 10.6 mm, BOZD of 6 mm and central thickness of 0.16 mm. The CRT lens is designed to be consistent with congruent anterior and posterior surfaces, and each surface consists of the following three zones: the central spherical zone, the mathematically designed sigmoidal cornea proximity the ‘return zone’ and the non-curving ‘landing area’. The lens also designs a convex elliptical edge terminal to smoothly connect the anterior and posterior surfaces.

The contact lenses are worn by three ophthalmologists. Participants were required to wear contact lenses for at least 8 consecutive hours per night. We plan to perform follow-up examinations at 1 month, followed by slit lamp examination and optometry every 3 months to monitor any potential adverse events.

### Dataset preparation

In this study, a total of 54 features were collected based on measurements obtained from the OPD Scan III instrument and Biometer. These measurements focused on higher-order aberrations (HOAs): total/corneal/internal: spherical aberration, coma aberration, trefoil, and total HOAs were collected under mesopic, 6 mm, and 4 mm pupil, respectively. In addition to higher-order aberrations, the comprehensive characteristics of the patients were also collected. Including age, gender, diopter, anterior corneal surface curvature, photopic/ mesopic pupil diameter, corneal thickness (CCT), lens thickness (LT), aqueous humor depth (AD), white to white distance (WTW), modulation transfer function (MTF) and other related parameters. The total sample size consisted of 225 participants, and these samples were divided into training and test sets in an 8:2 ratio to facilitate model development and evaluation.

Refraction: 0.5% tropicamide was used every 5 min for a total of 6 times after waiting 30 min so that the ciliary muscle is completely paralyzed to get accurate dilated pupils after the optometry results, such as the pupil return to the normal size of the pupil and then test with orthokeratology. The diopter was measured by a TOPCON KR-800 refractometer.

The corneal topography and wavefront aberration were analyzed by the OPDIII-SCAN corneal topography instrument (NIDEK, Japan). The Zernike polynomial is used to convert the corneal elevation contour data into corneal wavefront aberration data. The root mean square (RMS) values were used to calculate the RMS values under three different pupil diameters (4.0 mm, 6.0 mm, and mesopic pupil).

### Research roadmap

**Figure 1:**
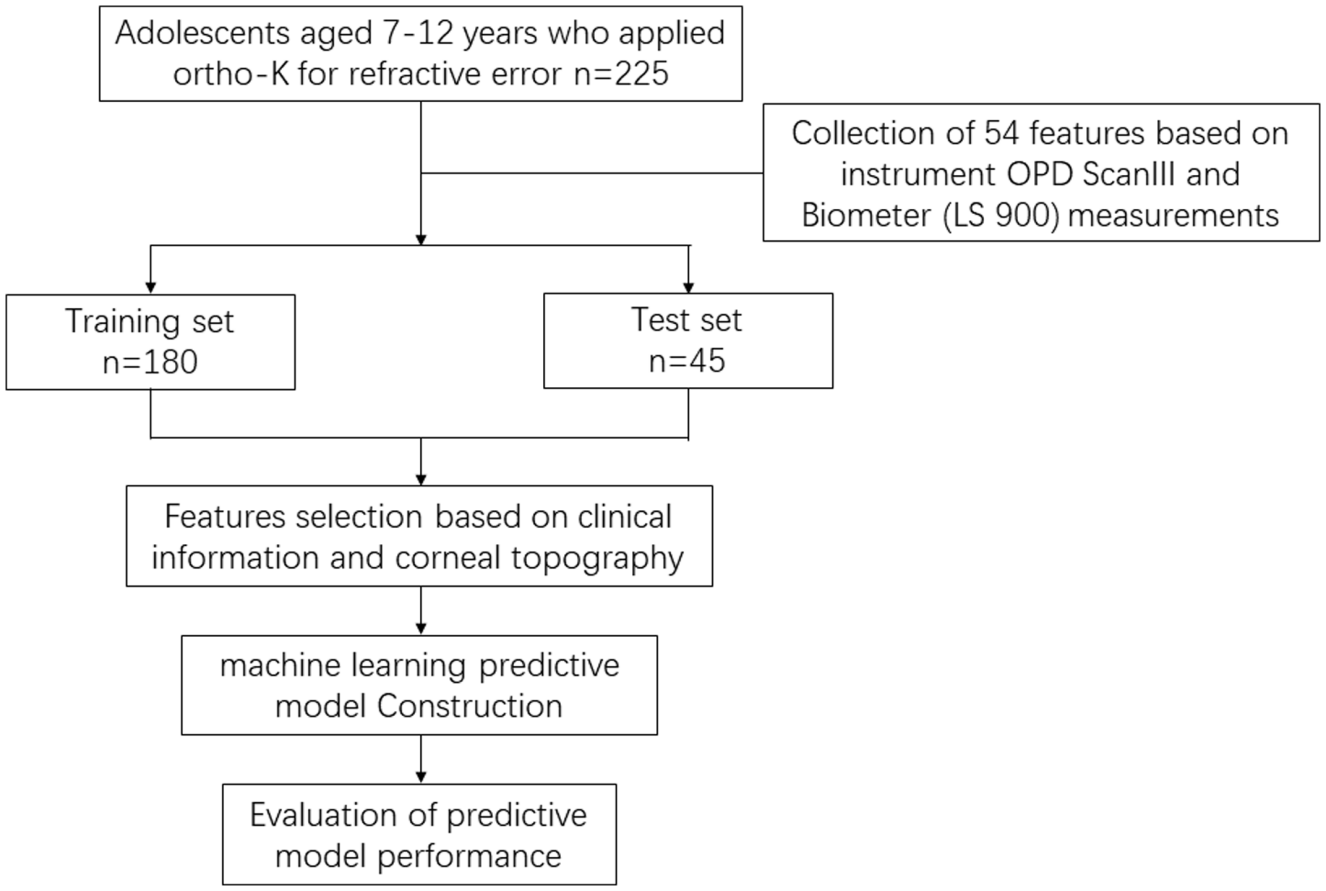
Shows the flow chart of the graphical description of the research design.

The research pipeline is shown in Figure 1.

### Feature selection

The Spearman algorithm is used to calculate the correlation between the features. We set the threshold to 0.9 and retain the features with a correlation coefficient less than 0.9 as the input features of the LASSO (Least Absolute Shrinkage and Selection Operator) regression model. Interestingly, the correlation coefficients of all the features generated in this step are below 0.9, so we incorporate all the features into further analysis. The LASSO regression model using the minimum absolute shrinkage and selection operator is applied to discover data sets to construct signatures. The LASSO regression model reduces the regression coefficient to zero by regularizing the weight *λ* and accurately sets the regression coefficients of many unrelated features to zero. To determine the optimal λ, a 10-fold cross-validation method is used to select the λ value with the smallest cross-validation error. We use the retained non-zero coefficient features to fit the regression model. LASSO regression modeling using Python scikit-learn package.

### Individualized prediction model building

After using Lasso regression for feature screening, the selected features are input into machine learning models such as K-Nearest Neighbor (KNN), Support Vector Machines (SVM), random forest (RF), Extra Tree, and eXtreme Gradient Boosting (XGBoost) to construct prediction models.

KNN is a supervised learning classification algorithm, which determines the nearest neighbor data points by comparing the distance, and then uses the K nearest sample points to determine the category of the data points to be classified, similar to the voting rule that the minority obeys the majority. It classifies the unknown samples and the K nearest neighbor samples into one category, and its prediction capability is excellent.

SVM is a kind of generalized linear classifier for binary classification of data. It operates according to the supervised learning method and is the linear classifier with the largest interval in the feature space. It is to find an optimal hyperplane to separate the different categories of samples. It maps the feature vector of the instance (taking two-dimensional as an example) to some points in the space. The purpose is to draw a line to ‘best’ distinguish the two types of points so that the line can also effectively classify any new points in the future. When linearly separable, the optimal classification hyperplane of two types of samples is found in the original space. When linearly inseparable, slack variables are added, and the samples in the low-dimensional input space are mapped to the high-dimensional space by using nonlinear mapping to make it linearly separable, so that the optimal classification hyperplane can be found in the feature space.

RF is an integrated algorithm composed of decision trees, and there is no correlation between different decision trees. Each decision tree is a classifier. Each data set randomly selects some features as input, which means that each sample will be judged and classified by each decision tree in the forest. Each decision tree will get a classification result. The classification with the most results is the final result of random forest.

Extra Trees is also an integrated model of decision trees, but it is more random than random forests. Each split uses a random threshold to select a subset for branch feature selection. It randomly selects a feature subset at each node and randomly splits to obtain the optimal branch attributes and branch thresholds. This randomness is conducive to creating more independent decision trees, reducing the model’s variance, and improving its performance.

XGBoost is a machine-learning algorithm based on a gradient boosting tree. It combines the gradient boosting framework and the decision tree model, and gradually improves the prediction performance by iteratively training a series of decision trees. It uses the addition model to construct multiple base learners and learns the deviation between the results of the previous base learners and the true value. XGBoost continuously reduces the difference between model values and actual values through the learning of multiple learners. The prediction result of the final model is the sum of the prediction results of all base learners. At the same time, XGBoost can optimize the loss function and minimize the error between the predicted value and the actual value.

### Performance evaluation of predictive models

In order to evaluate the performance of the model, we considered the accuracy and area under the curve (AUC) of the training set and the test set. In addition, we used decision curve analysis (DCA) to evaluate the clinical utility of the model.

AUC is the area under the ROC curve, which uses the positive correlation law to convert the predicted control effectiveness into the predicted probability. Then a series of true positive rates (TPR) and false positive rates (FPR) can be obtained and used to construct the receiver operating characteristic curve (ROC). The AUC is derived from the area between the ROC curve and the FPR axis, the abscissa axis. TPR was defined as sensitivity and FPR was defined as 1-specificity. In terms of performance evaluation, the externally validated AUC is often used as an indicator of the performance of ensemble learning systems. The closer to 1, the better the performance of the classifier it represents.

Confusion matrix is a summary table used to evaluate the performance of classification models in machine learning. It presents the results predicted by the classification model in the form of a matrix, in which the records in the data set are summarized by their real and predicted class labels. The rows of the matrix represent the true value and the columns represent the predicted value. The confusion matrix can help us to evaluate the prediction performance of different models.

In addition, we also use Decision Curve Analysis (DCA) to evaluate the clinical practicability of the model. The DCA curve is the calculation of the clinical ‘net benefit’ of one or more predictive models or diagnostic tests compared to the default strategy of treating all patients or untreated patients. The net benefit is calculated within a range of threshold probabilities, which are defined as the minimum probability of disease requiring further intervention. Net benefit = sensitivity × prevalence- (1-specificity) × (1-prevalence) × w, where w is the ratio of threshold probabilities. For the prediction model that gives the disease prediction probability p, the sensitivity and specificity under a given threshold probability pt. are calculated by defining the test positive as p ≥ pt.

### Statistical methods

All of these operations are implemented using Python version 3.9.

## Results

### Feature selection and prediction model building

**Figure 2:**
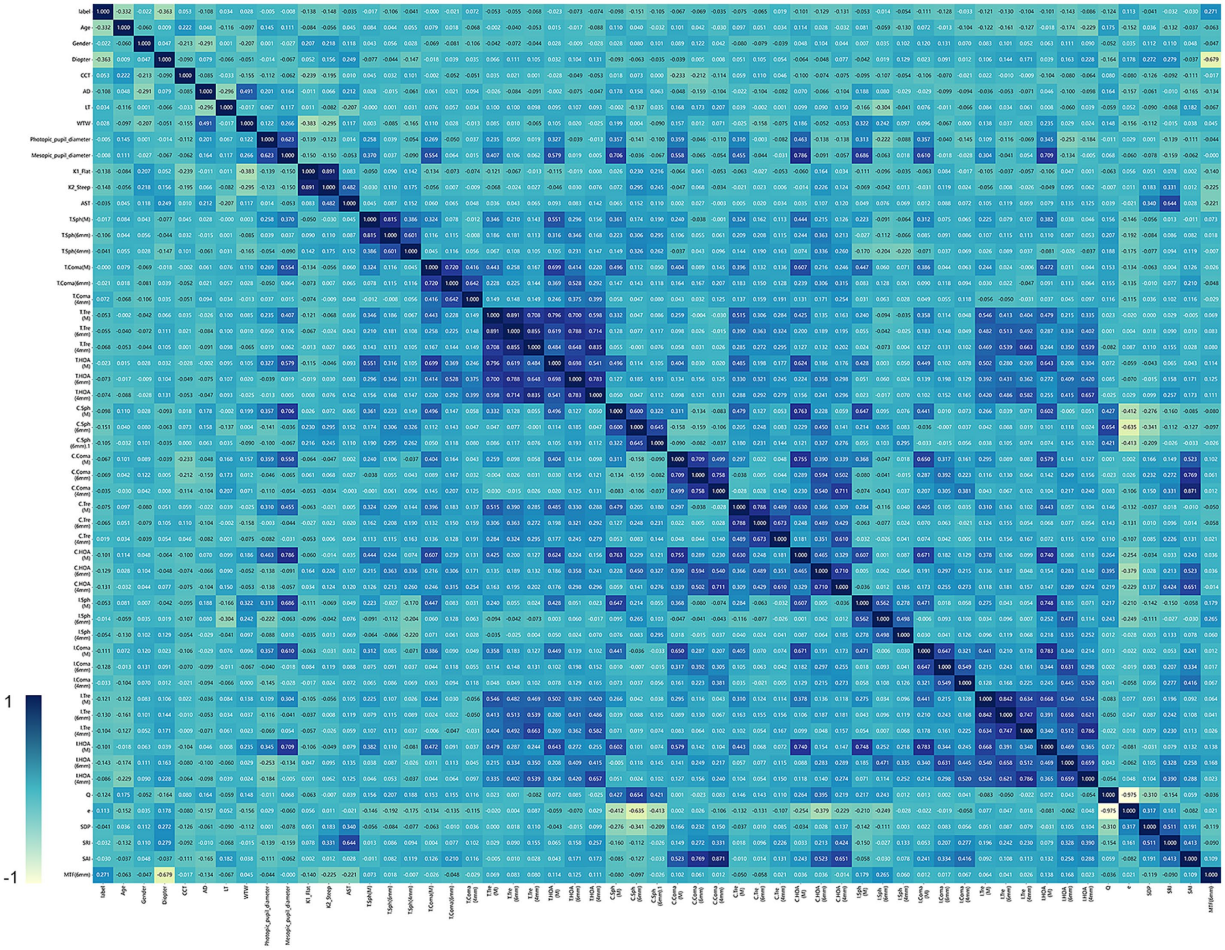
The heat map shown in figure illustrates the correlation between different clinical features. The color gradient in the heat map represents the intensity of the correlation. Specifically, the bluer the color, the stronger the positive correlation, indicating that the variables tend to increase or decrease together. On the other hand, the yellower the color, the weaker the correlation or the negative correlation.

A total of 54 features were collected from a sample of 225 cases in this study, and Table 1 shows the descriptive analysis of all the collected features. Heatmap shows the correlations between each clinical feature, it is indicated that Long Diameter, Short Diameter, and Diameter have maximum correlation coefficient (Figure 2). The depth of the color block represents the correlation between the two features. The closer the correlation coefficient is to 1, the deeper the blue color will appear, while the closer it is to 0, the lighter yellow the color will be. The algorithm removed some features with low correlation to the predicted outcome.

**Table 1:** Basic statistical information of all the features in this study.

Feature	Mean	SE	*M*	SD	Var	Range
Age	9.182	0.096	9	1.442	2.078	7–12
Gender	1.524	0.033	2	0.501	0.251	1–2
Diopter	2.566	0.083	2.25	1.239	1.535	0.5–6.25
Pre-AL	24.469	0.052	24.49	0.780	0.608	22.38–26.75
AL-6M	24.610	0.051	24.68	0.767	0.588	22.54–26.78
AL-1Y	24.762	0.051	24.85	0.768	0.589	22.59–26.81
CCT	549.044	2.090	550	31.357	83.275	474–627
AD	3.234	0.015	3.26	0.219	0.048	2.63–3.82
LT	3.366	0.017	3.38	0.262	0.068	0.39–3.87
WTW	12.071	0.027	12.08	0.398	0.159	11.25–13.64
Photopic pupil diameter	4.288	0.064	4.05	0.958	0.917	2.79–7.66
Mesopic pupil diameter	6.641	0.049	6.61	0.736	0.541	4.63–8.74
K1 Flat	42.842	0.074	42.78	1.114	1.241	39.02–46.3
K2 Steep	44.075	0.088	44.06	1.316	1.732	39.66–47.14
AST	1.235	0.040	1.14	0.603	0.363	0.1–3.1
T.Sph (M)	0.159	0.009	0.118	0.133	0.018	0.002–0.824
T.Sph (6 mm)	0.109	0.005	0.094	0.077	0.006	0.006–0.4
T.Sph (4 mm)	0.030	0.002	0.023	0.036	0.001	0–0.46
T.Coma (M)	0.268	0.012	0.226	0.185	0.034	0.017–1.309
T.Coma (6 mm)	0.180	0.006	0.162	0.097	0.009	0.031–0.597
T.Coma (4 mm)	0.056	0.002	0.051	0.030	0.001	0.007–0.204
T.Tre (M)	0.320	0.013	0.284	0.197	0.039	0.042–0.99
T.Tre (6 mm)	0.249	0.010	0.216	0.143	0.021	0.028–0.805
T.Tre (4 mm)	0.111	0.005	0.094	0.076	0.006	0.011–0.54
T.HOA (M)	0.548	0.019	0.492	0.284	0.081	0.129–1.748
T.HOA (6 mm)	0.399	0.011	0.365	0.169	0.029	0.166–1.323
T.HOA (4 mm)	0.161	0.006	0.139	0.090	0.008	0.009–0.636
C.Sph (M)	0.370	0.014	0.322	0.207	0.043	0.052–1.183
C.Sph (6 mm)	0.241	0.007	0.235	0.101	0.010	0.047–0.712
C.Sph (4 mm)	0.051	0.002	0.048	0.032	0.001	0.004–0.3
C.Coma (M)	0.358	0.018	0.271	0.269	0.072	0.019–1.679
C.Coma (6 mm)	0.230	0.009	0.21	0.130	0.017	0.019–0.678
C.Coma (4 mm)	0.066	0.003	0.059	0.042	0.002	0.01–0.335
C.Tre (M)	0.244	0.012	0.203	0.182	0.033	0.034–1.218
C.Tre (6 mm)	0.170	0.006	0.155	0.094	0.009	0.029–0.573
C.Tre (4 mm)	0.062	0.003	0.054	0.039	0.002	0.006–0.312
C.HOA (M)	0.636	0.023	0.563	0.339	0.115	0.143–2.283
C.HOA (6 mm)	0.419	0.009	0.381	0.137	0.019	0.196–1.199
C.HOA (4 mm)	0.120	0.003	0.11	0.049	0.002	0.042–0.459
I.Sph (M)	0.347	0.015	0.301	0.225	0.051	0.025–1.5
I.Sph (6 mm)	0.199	0.008	0.182	0.120	0.014	0.015–1.107
I.Sph (4 mm)	0.036	0.002	0.031	0.026	0.001	0.002–0.208
I.Coma (M)	0.370	0.015	0.316	0.230	0.053	0.061–1.34
I.Coma (6 mm)	0.247	0.008	0.227	0.122	0.015	0.048–0.853
I.Coma (4 mm)	0.073	0.003	0.065	0.038	0.001	0.01–0.323
I.Tre (M)	0.272	0.013	0.228	0.192	0.037	0.037–1.331
I.Tre (6 mm)	0.209	0.009	0.171	0.138	0.019	0.019–0.725
I.Tre (4 mm)	0.097	0.005	0.078	0.071	0.005	0.005–0.459
I.HOA (M)	0.680	0.023	0.596	0.350	0.123	0.163–2.011
I.HOA (6 mm)	0.457	0.014	0.396	0.206	0.043	0.204–1.647
I.HOA (4 mm)	0.166	0.006	0.138	0.090	0.008	0.075–0.619
*Q*	−0.195	0.011	−0.21	0.158	0.025	−0.85-0.4
*e*	0.400	0.018	0.45	0.263	0.069	−0.63-1.83
MTF (6 mm)	0.167	0.002	0.159	0.026	0.001	0.137–0.328

**Figure 3:**
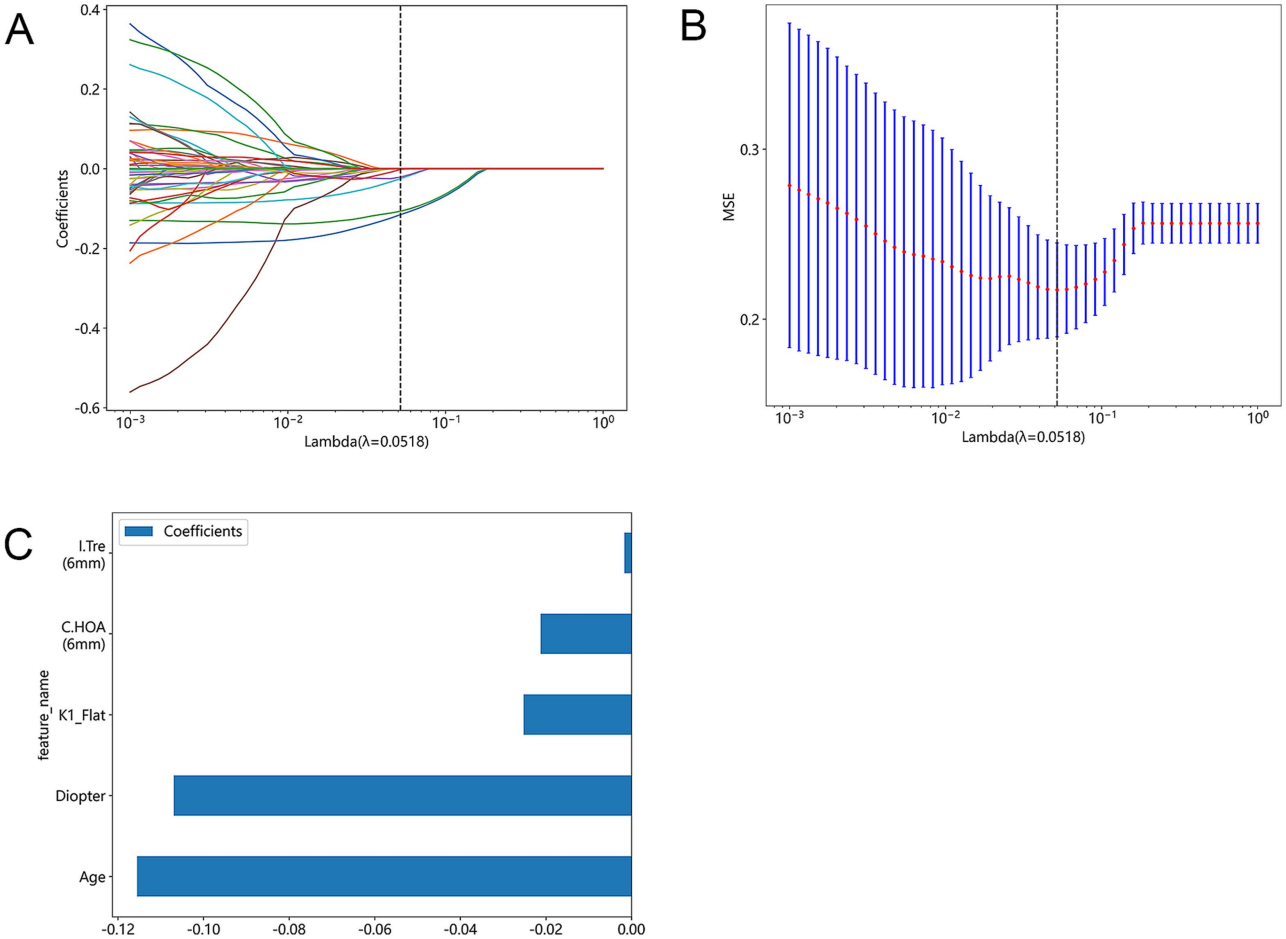
Lasso feature selection: Select non-zero coefficients to build a logistic regression model with minimum absolute shrinkage and selection operator (Lasso). **(A)** Shows the coefficients obtained by Lasso feature selection using a 10-fold validated logistic regression model. The coefficient represents the strength and direction of the relationship between each feature and the result variable. **(B)** Shows the average standard error results obtained in the Lasso feature selection process when lambda is set to 0.0518(MSE). **(C)** Shows a bar graph that shows all the features used in the Lasso feature selection process and their corresponding *p*-values. The *p*-value represents the significance level of each feature associated with the outcome variable.

Based on the results obtained by the Lasso method, we were able to reduce the total number of features (including basic clinical features and corneal topography) of the original cohort to five potential predictors, as shown in Figure 3C. These predictors included age, diopter, flat keratometry (K), corneal higher-order aberrations (6 mm) (C.HOA 6 mm), and intraocular trefoil (6 mm) (I.Tre 6 mm). The model incorporating these predictors can achieve a minimum mean square error (MSE), as shown in Figure 3B. All variables showed non-zero coefficients in the Lasso linear regression model, and the *p*-values were all less than 0.05. Figure 3A shows the 10-fold validation coefficient and the mean standard error (MSE). These weights come from clinical variables. The ensemble learning system can determine the weight of different features based on the analysis of all samples. For a visual representation of the weights assigned to different features, see Figure 3C.

### Performance evaluation of predictive models

**Figure 4:**
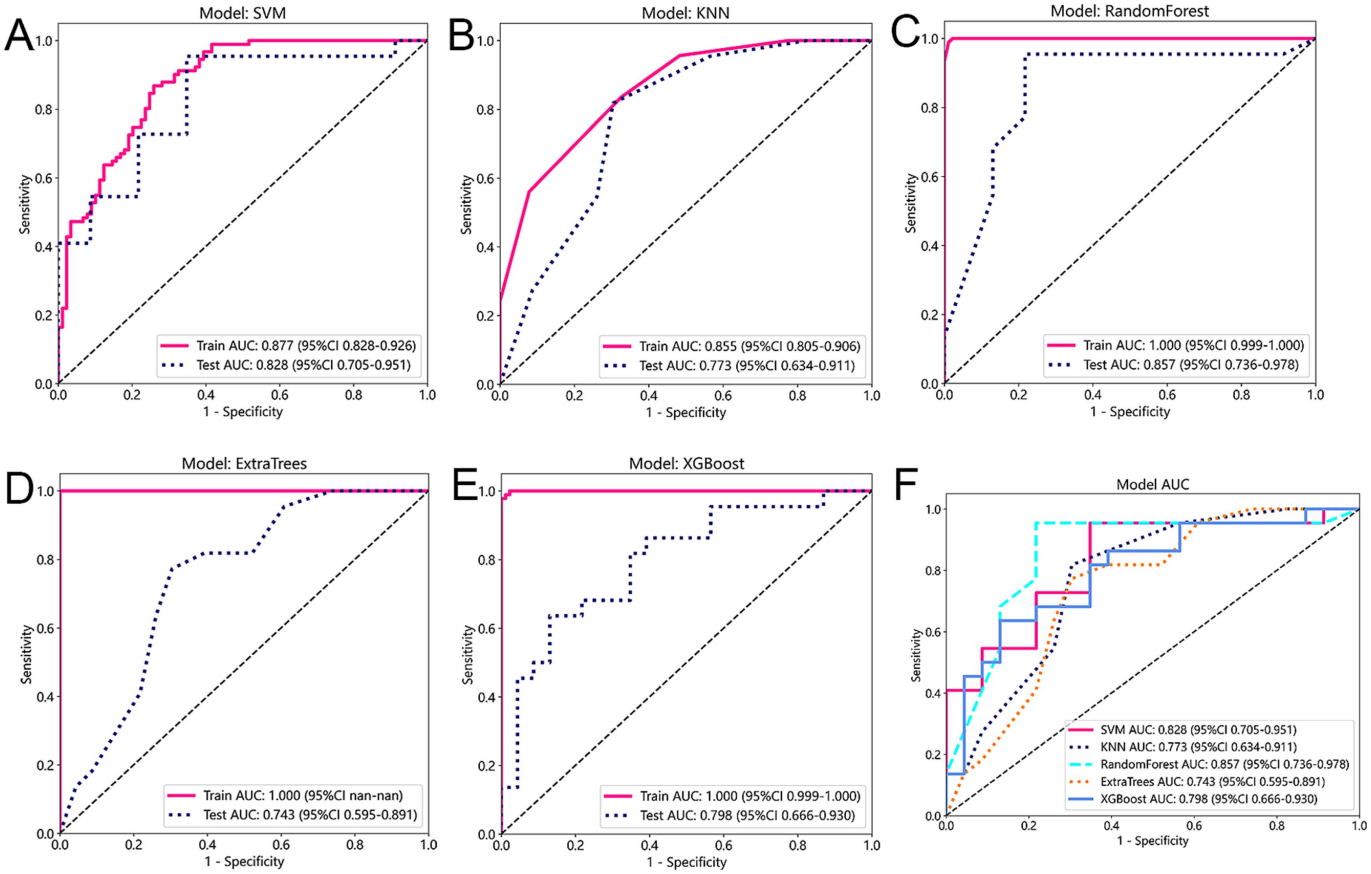
**(A–E)** This figure provides a comparison of five different prediction models: Support Vector Machines (SVM), K-Nearest Neighbors (KNN), Random Forest, Extra Trees, and XGBoost. **(F)** The figure displays the AUC (Area Under the Curve) values for each model on both the training and test cohorts.

In order to transform predictive control validity into predictive probability, we apply the law of positive correlation. This allowed us to obtain a series of true positive rates (TPR) and false positive rates (FPR), which were then used to construct the receiver operating characteristic curve (ROC). The area under the ROC curve (AUC) was calculated as the area between the ROC curve and the FPR (false positive rate) axis. Based on the evaluation indexes of these models, five models with the best performance are selected, which are SVM, KNN, RF, Extra Trees and XGBoost. In terms of performance evaluation, the externally verified AUC is used as an indicator of the performance of the ensemble learning system. In Figure 4, we compare the AUC of various prediction models on the training set and test set. The AUC value of the SVM model reached 0.877 in the training cohort and 0.828 in the test cohort. The AUC value of the KNN model was 0.855 in the training cohort and 0.773 in the test cohort. The AUC value of the RF model was 1.000 in the training cohort and 0.857 in the test cohort. The AUC value of the Extra Trees model was 1.000 in the training cohort and 0.773 in the test cohort. The AUC value of the KNN model was 0.855 in the training cohort and 0.773 in the test cohort. The ROC curves of the test queues of the five models are shown in the figure. Taking into account the unity of the training and test queues and the AUC value, the SVM model performs best.

**Figure 5:**
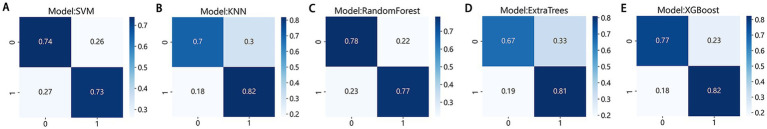
**(A–E)** The confusion matrix of the five prediction models is used to demonstrate their predictive ability.

Confusion matrix is a valuable tool for evaluating the performance of classification models in machine learning. It compares real and predicted class labels in a matrix format, thus providing a summary of model predictions. The rows of the matrix represent the true values, and the columns represent the predicted values. By analyzing the confusion matrix, we can evaluate the prediction performance of different models. We observed that all the prediction models performed well. The true positive rate and true negative rate of XGBoost are 0.77 and 0.83, respectively (Figure 5). These values indicate the ability of the model to correctly identify positive and negative samples.

**Figure 6:**
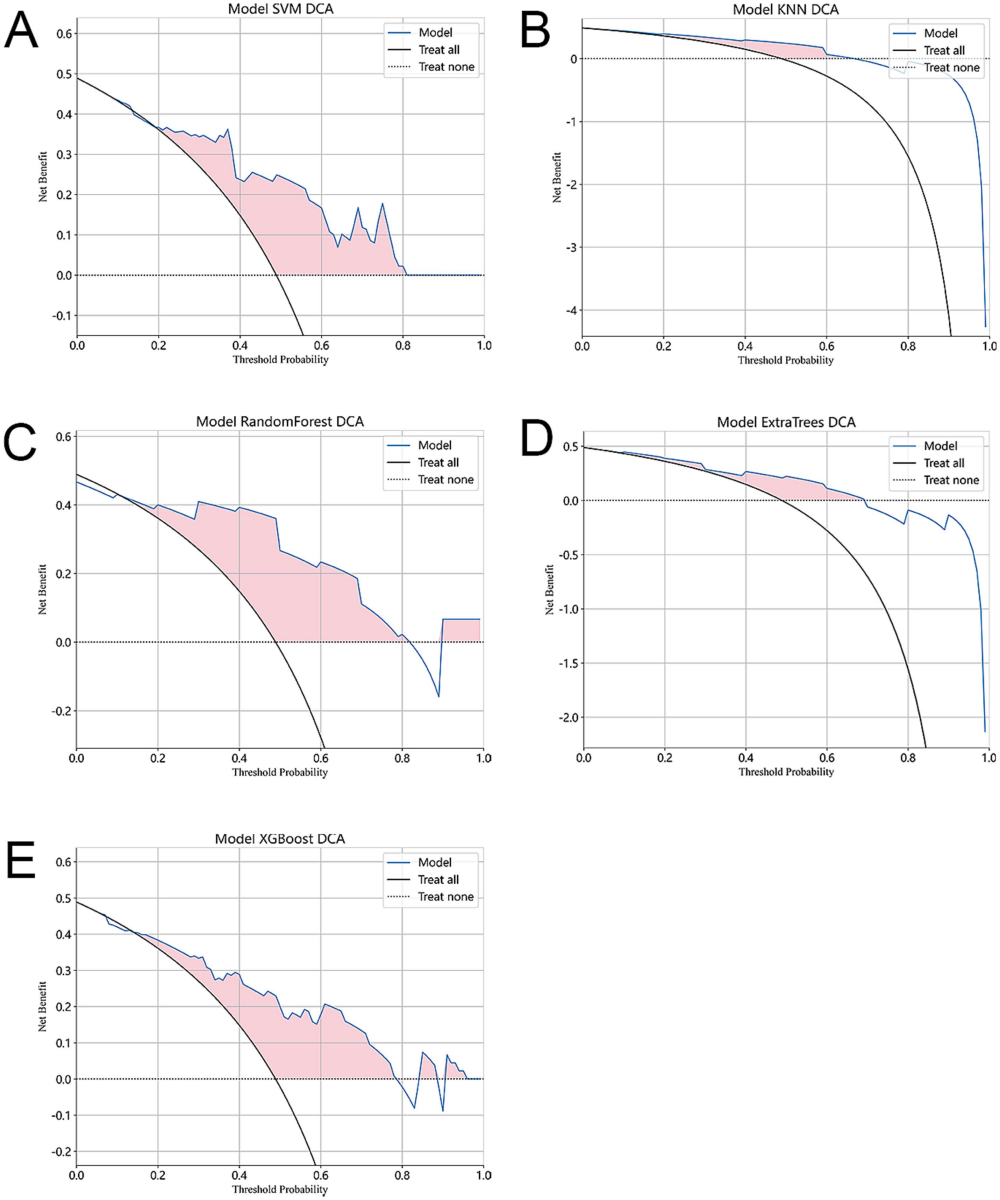
In our evaluation, we use decision curve analysis (DCA) to evaluate the performance of each model. **(A–E)** The figure shows the results of DCA. The x-axis of the graph represents the threshold probability, indicating the probability of considering treatment intervention. The y-axis represents the net benefit and quantifies the clinical utility of the model. The figure shows that compared with the absence of any predictive model (i.e., the use of full treatment or no treatment regimen), all five models showed significant benefits of interventions in patients with predictive probabilities. This shows that the use of prediction models can improve the accuracy of treatment decisions. In addition, the SVM model showed the highest clinical benefit.

In addition to using the confusion matrix evaluation model, this study also included decision curve analysis (DCA). The x-axis of the DCA diagram corresponds to the threshold probability, while the y-axis represents the net income. Figure 6 illustrates the decision curve analysis of the five models. Interestingly, all five models showed that interventions had significant benefits in patients with predictive probabilities compared to those without predictive models (such as full treatment or no treatment regimen). Among them, the SVM and RF model showed greater clinical benefits in terms of net benefits. This shows that the SVM and RF model can provide patients with more effective intervention or treatment decisions based on the patient’s predictive probability.

**Figure 7:**
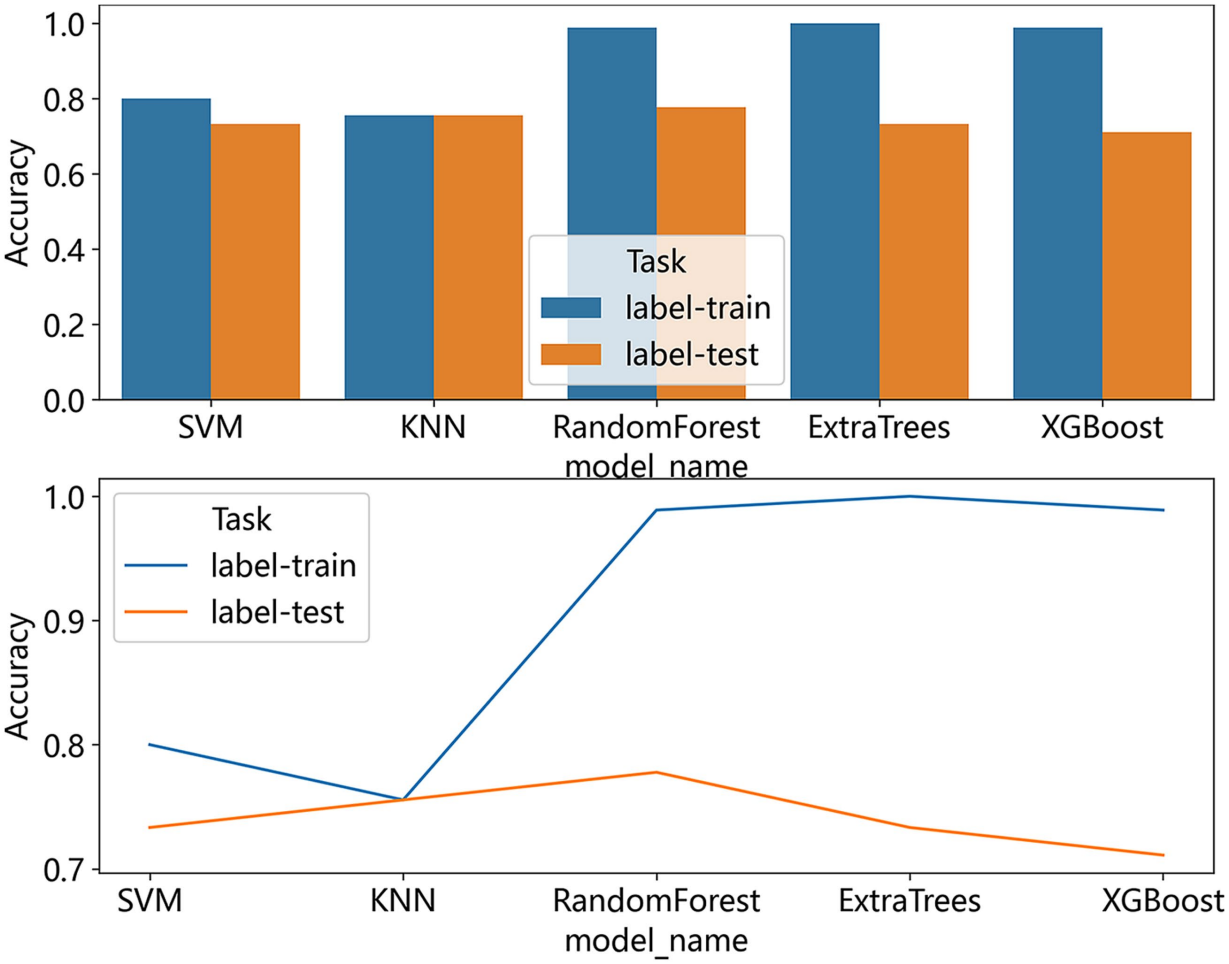
The bar graphs and line chart show a comparison of the accuracy of all the models we used to predict the effect of ortho-k on myopia control.

We also compared the accuracy of the five models, as shown in Figure 7, the KNN model showed the most consistency in accuracy between the training set and the test set, and the RF model performed the best in terms of consistency and accuracy.

Based on the analysis of this study, among these models, the SVM model has good predictive ability and is the most reliable and accurate model to predict the myopia control effect of ortho-k.

## Discussion

Ortho-k has become popular among parents as a more effective measure to slow myopia progression. However, studies have found that there are still some patients using ortho-k to control myopia is not effective (17). Moreover, it usually takes 1–2 years of follow-up duration to evaluate ortho-k efficiency and determine whether the treatment has successfully slowed myopia progression (11). In the health system, the prediction model is an effective tool for risk assessment, decision-making, and benefit evaluation. In our study, we developed and internally validated five prediction models by non-invasive examinations to predict the control effect of ortho-k, and it can provide a reference for ophthalmologists to determine whether the child is suitable for ortho-k to control myopia, to improve the satisfaction of children and their parents.

Considering that the current examination for ortho-k in our hospital has been very comprehensive, we proposed to construct multi-feature clinical prediction models to predict ortho-k efficacy by retrospectively analyzing these data. The models established in this study exhibited great predictive performance in both the training set and the test set, based on the AUC and DCA curves. This indicates that the models can accurately predict the therapeutic effect of ortho-k. In the process of model development, combined with mRMR and LASSO methods, the initial 54 candidate features are reduced to 5 features. These characteristics, including age, diopter, flat keratometry, corneal higher-order aberrations, and internal trefoil aberration, were found to be associated with ortho-k effect.

In previous studies, the effect of ortho-k may have been different for patients of different ages (5). Wang et al. proved that younger individuals have better myopia control (18), which may be because younger children show faster axial growth, so they can benefit more from early ortho-k intervention (19). However, other studies have shown that better myopia control occurs at older ages. It is difficult to prove whether this is due to the ortho-k effect or if it occurs naturally as children age and myopia progresses later in life due to slower axial elongation (20).

The initial diopter is also supported by studies as a differential feature. Fu et al. proposed that a higher baseline spherical equivalent may provide an advantage for ortho-k by reducing axial growth and effectively controlling myopia (21). This may be due to the large corneal steepness in the middle and periphery of high myopia, and the resulting peripheral retinal defocus, which leads to the deceleration of axial growth. However, contrary to these findings, some studies did not observe a significant correlation between initial diopter and axial changes (5). Given these conflicting results, further prospective studies are needed to fully investigate the relationship between these two variables.

Studies have shown that ortho-k can make the central cornea flatter and the paracentral cornea steeper. The heterogeneity of corneal morphological changes may affect the peripheral refractive distribution, which is considered to be related to myopia control (22). The main mechanism for ortho-k to reduce myopia is to flatten the curvature of the anterior surface of the cornea (23). It is worth noting that the steeper cornea may lead to a smaller central corneal flattening area after treatment (20). However, it should be emphasized that there is no study to determine the direct relationship between pre-treatment anterior corneal curvature and myopia control.

Higher-order aberrations (HOAs) are optical defects that cannot be corrected by conventional sphero-cylinder lenses (24). Animal studies have shown that higher-order aberrations can reduce the quality of retinal images and cause changes in the intensity of the light at the pupil of the eye. These aberrations provide optical signals that help regulate the growth of the eyeball and refractive error development (10). A study by Liu et al. found that after controlling for age, baseline diopter and other known factors affecting axial elongation, the spherical aberration and HOA RMS values in eyes with slower axial development were higher, indicating that higher-order aberrations were negatively correlated with myopia progression (25). Another study showed that the control of myopia by ortho-k increased the Zernike coefficient, thus increasing the RMS value of the total higher-order aberration range in children. However, it is also found that the visual quality decreases after wearing the lens, which is manifested by the increase of higher-order aberrations such as spherical aberration, horizontal coma vertical coma, and MTF equivalent value (26). These changes usually stabilize after a month (27).

The relationship between higher-order aberrations and axial growth before ortho-k has not been widely studied. The Lasso regression screening of corneal HOAs and intraocular trefoil at 6 mm pupil diameter as predictors of decreased axial elongation is a more groundbreaking finding and may provide a reference for future research directions.

We found that most studies were prospective longitudinal studies, and the selection of variables by different researchers was based on empirical and scientific assumptions, often resulting in incomplete selection and opposite results. There are also fewer studies on the correlation between eye axial elongation and ortho-k pre-treatment indicators and multiparametric modal prediction models for ophthalmic use have been explored in a series of studies (28). Our multi-feature prediction model provides a comprehensive and objective analysis method. It shows great prediction accuracy on both the training set and the test set. In addition, the results of this study lay the foundation for the design of more extensive longitudinal studies.

Nevertheless, we must admit that our research has some limitations. First of all, this is a retrospective study conducted only within our institution and it should be noted that our sample size is small, suggesting that prospective studies with larger sample sizes are needed in the future to provide more reliable evidence. In addition, external validation is required in other centers to confirm and verify the performance of the model. In addition, some factors related to the progression of myopia, such as parents’ myopia status and outdoor time, should also be taken into account. However it is difficult to standardize and unify the time of different sports and intensity in outdoor sports, and also difficult to count the myopia of parents, so the above two characteristics are not included in the study. These features can be explored in future studies to better understand their impact and significance on myopia.

## Conclusion

In conclusion, we developed and validated several prediction models of the effect of individualized prediction of myopia control efficacy treated with overnight ortho-k based on clinical and corneal topography features which can predict the efficacy of ortho-k before prescribing ortho-k, which is to provide a reference for ophthalmologists to determine whether children are suitable for ortho-k to control myopia, and can also improve patient satisfaction. Optimizing the optical design of the higher-order aberrations of the ortho-k lens can improve the myopia control effect without significantly affecting the visual function. This study may provide designers with directions to optimize lens design.

## Data Availability

The original contributions presented in the study are included in the article/Supplementary material, further inquiries can be directed to the corresponding author/s.
