# Supplementary material for: Development and evaluation of machine learning models for individualized prediction of myopia control efficacy treated with overnight orthokeratology
Source: Front Med (Lausanne). 2025 May 12;12:1559435. doi: 10.3389/fmed.2025.1559435 (PMC12104297; doi:10.3389/fmed.2025.1559435)
Supplement: Supplementary file 1 [file Data_Sheet_1.pdf]

Patient ID	Age	Gender	Diopter	Pre-AL	AL-6M	AL-1Y	1Y/P	label
65	11	1	3.75	26.18	26	26.04	0.994652406	0
109	9	2	5.5	25.28	25.16	25.15	0.994857595	0
223	8.5	2	3.5	23.94	23.85	23.85	0.996240602	0
133	9	2	2.75	24.51	24.42	24.42	0.996328029	0
64	11	1	3.75	26.34	26.23	26.25	0.996583144	0
2	12	2	3.5	24.81	24.67	24.74	0.997178557	0
46	7	1	3	25.22	25.23	25.16	0.997620936	0
193	8	2	3.75	22.92	22.77	22.87	0.997818499	0
16	12	1	4.5	25.9	25.85	25.85	0.998069498	0
204	10	2	3.5	23.86	23.78	23.82	0.998323554	0
1	12	2	4.75	25.12	25.05	25.08	0.998407643	0
14	10	2	2.75	24.33	24.34	24.31	0.99917797	0
47	7	1	2.5	24.89	24.85	24.87	0.999196464	0
110	9	2	5.25	24.98	24.93	24.96	0.999199359	0
15	12	1	3.5	25.41	25.4	25.39	0.999212908	0
134	10	2	5	25.48	25.52	25.46	0.999215071	0
188	10	2	5.5	25.97	25.89	25.97	1	0
135	10	2	4.75	25.38	25.45	25.39	1.000394011	0
35	8	1	1.75	24.13	24.12	24.15	1.000828844	0
89	11.5	2	2	25.07	25.05	25.1	1.001196649	0
112	9	2	2.5	23.97	24.05	24	1.001251564	0
138	8	2	1.5	22.81	22.82	22.84	1.001315213	0
150	12	1	5.75	26.31	26.36	26.35	1.001520334	0
189	10	2	4	25.99	25.97	26.03	1.001539053	0
37	9	2	4.25	25.72	25.74	25.76	1.00155521	0
90	11.5	2	2.5	25.36	25.35	25.4	1.001577287	0
171	14	1	2.25	25.07	25.06	25.11	1.001595533	0
185	9	1	2	24.83	24.77	24.87	1.001610954	0
191	12	2	1.25	24.15	24.14	24.2	1.002070393	0
209	12	1	6.25	26.75	26.78	26.81	1.002242991	0
83	9	2	4.5	24.72	24.7	24.78	1.002427184	0
180	13	2	2.5	24.37	24.33	24.43	1.002462043	0
104	10	1	2	24.88	24.9	24.95	1.002813505	0
213	9	1	2.75	23.47	23.47	23.55	1.003408607	0
123	9	1	4	26.07	26.15	26.16	1.003452244	0
10	12	2	5.25	24.86	24.91	24.95	1.003620274	0
214	9	1	3	23.51	23.51	23.6	1.003828158	0
111	10	2	2	24.1	24.12	24.2	1.004149378	0
186	10	2	3	23.71	23.69	23.81	1.00421763	0
19	8	1	3.5	24.62	24.68	24.73	1.004467912	0
124	9	1	3.5	24.51	24.65	24.62	1.004487964	0
190	9	1	2	24.49	24.49	24.61	1.004899959	0
105	9	2	2.5	24.39	24.44	24.51	1.004920049	0
116	7	1	3.25	24.39	24.45	24.51	1.004920049	0
13	10	2	3	24.25	24.35	24.37	1.004948454	0
96	10	2	1.75	24.19	24.21	24.31	1.004960728	0
151	12	1	5.25	26.16	26.29	26.3	1.005351682	0
122	9	1	3.5	25.75	25.85	25.89	1.005436893	0

224	8.5	2	1.75	23.52	23.56	23.65	1.005527211	0
91	11	2	2.25	24.81	24.85	24.95	1.005642886	0
179	13	2	2	24.27	24.31	24.41	1.005768438	0
20	8	1	3	24.65	24.68	24.8	1.006085193	0
173	8	1	3.75	24.65	24.64	24.8	1.006085193	0
117	7	1	3.25	24.2	24.29	24.35	1.006198347	0
127	9	2	5.25	24.88	25.08	25.04	1.006430868	0
205	10	1	1.5	24.46	24.57	24.62	1.006541292	0
132	9	2	2.75	24.4	24.45	24.56	1.006557377	0
192	8	2	3.75	22.75	22.82	22.9	1.006593407	0
225	10	1	2.25	25.7	25.77	25.87	1.006614786	0
187	10	2	3.5	23.97	23.93	24.13	1.00667501	0
137	8	1	3.25	25.37	25.41	25.54	1.006700828	0
76	11	2	1	23.3	23.27	23.46	1.006866953	0
129	9	1	4.25	26.14	26.21	26.32	1.006885998	0
131	10	2	1.5	23.79	23.88	23.96	1.00714586	0
208	12	1	2.75	25.51	25.62	25.7	1.00744806	0
113	8	2	1.5	23.8	23.91	23.98	1.007563025	0
9	12	2	5	24.86	25.01	25.05	1.0076428	0
201	14	1	3	24.82	24.9	25.01	1.007655117	0
54	8	1	2.75	24.76	24.68	24.95	1.007673667	0
160	11	2	2.75	24.55	24.68	24.74	1.007739308	0
161	11	2	2.75	24.59	24.71	24.79	1.008133388	0
149	10.5	1	0.75	23.91	24.1	24.11	1.008364701	0
61	11	1	2.5	25.04	25.09	25.25	1.008386581	0
26	10	2	2.5	24.63	24.8	24.84	1.008526188	0
36	9	2	4.5	25.75	25.9	25.97	1.008543689	0
92	11	2	2.25	24.77	24.8	24.99	1.008881712	0
126	9	2	5	24.67	24.79	24.89	1.008917714	0
121	7	2	3	25.57	25.69	25.8	1.008994916	0
119	11	1	2	24.45	24.63	24.67	1.008997955	0
176	11	1	0.5	23.89	24	24.11	1.009208874	0
145	9.5	2	1	22.73	22.89	22.94	1.009238891	0
128	9	1	4	25.88	26	26.12	1.00927357	0
118	11	1	2.75	24.77	24.94	25	1.009285426	0
93	7	1	2	23.65	23.71	23.87	1.009302326	0
139	8	2	0.5	22.38	22.54	22.59	1.009383378	0
159	9	1	1.75	24.36	24.49	24.59	1.009441708	0
200	14	1	3.5	24.95	25.09	25.19	1.009619238	0
84	9	2	4	24.81	24.9	25.05	1.009673519	0
55	8	1	2	24.72	24.68	24.96	1.009708738	0
43	12	1	4.5	24.63	24.69	24.87	1.009744214	0
106	9	2	2.5	24.58	24.67	24.82	1.009764036	0
120	7	2	2.5	25.27	25.44	25.52	1.009893154	0
175	11	1	2.75	24.62	24.77	24.87	1.010154346	0
212	10	1	1.25	24.08	24.1	24.33	1.01038206	0
33	8	2	2	24.03	24.24	24.28	1.010403662	0
184	8	1	2.5	24.01	24.04	24.26	1.010412328	0
148	10.5	1	0.75	23.93	24.06	24.18	1.010447137	0
25	10	2	2.25	24.68	24.85	24.94	1.010534846	0
222	8	2	3.75	24.14	24.28	24.4	1.010770505	0

95	10	2	2.25	24.26	24.4	24.53	1.011129431	0
130	10	2	2.25	24.23	24.3	24.5	1.011143211	0
63	8	2	4.25	24.87	25.05	25.15	1.011258544	0
58	10	2	4.75	24.81	24.86	25.09	1.011285772	0
172	8	1	3.75	24.81	24.92	25.09	1.011285772	0
28	8	2	3	25.48	25.66	25.77	1.011381476	0
202	9	1	2.75	25.26	25.32	25.55	1.011480602	0
158	9	1	1.75	24.37	24.57	24.65	1.011489536	0
21	9	2	1	23.41	23.6	23.68	1.011533533	0
177	9	1	2.5	24.24	24.44	24.52	1.011551155	0
196	7.5	2	2	23.32	23.5	23.59	1.011578045	0
5	12	1	2.5	24.96	25.05	25.25	1.01161859	0
6	12	1	2.25	24.79	24.88	25.08	1.011698265	0
147	9	2	1	23.72	23.98	24	1.011804384	1
31	11	1	1.25	23.55	23.76	23.83	1.011889597	1
108	9	2	0.75	23.55	23.59	23.83	1.011889597	1
94	7	1	2.25	23.38	23.52	23.66	1.011976048	1
103	10	1	2.25	25.04	25.1	25.34	1.011980831	1
99	8.5	2	2	24.16	24.4	24.45	1.012003311	1
153	8	2	4	24.99	25.29	25.29	1.012004802	1
27	8	2	3.25	25.53	25.61	25.84	1.012142577	1
168	10	1	1.25	24.43	24.52	24.73	1.012279984	1
144	9.5	2	1	22.65	22.89	22.93	1.012362031	1
34	8	1	2	24.25	24.51	24.55	1.012371134	1
167	10	1	2	24.48	24.61	24.79	1.012663399	1
62	8	2	4.5	24.81	25.03	25.13	1.012898025	1
78	8	1	4.5	24.6	24.82	24.92	1.01300813	1
181	9	1	2.5	23.79	23.95	24.1	1.013030685	1
39	8	2	2.25	23.71	24.06	24.02	1.013074652	1
203	9	1	2.75	25.2	25.31	25.53	1.013095238	1
146	9	2	0.5	23.58	23.87	23.89	1.013146735	1
195	7.5	2	1.5	23.43	23.52	23.74	1.013230901	1
51	10	2	1.25	24.07	24.27	24.39	1.013294558	1
40	10	2	1.75	23.06	23.25	23.37	1.013443192	1
210	9	1	0.75	24.53	24.62	24.86	1.013452915	1
125	9	1	3.5	24.49	24.73	24.82	1.013474888	1
41	10	2	1.5	22.99	23.21	23.3	1.013484124	1
77	8	1	2.25	24.31	24.55	24.64	1.013574661	1
183	8	1	3.25	24.31	24.43	24.64	1.013574661	1
152	8	2	4.25	24.92	25.28	25.26	1.01364366	1
174	9	1	1.25	23.23	23.39	23.55	1.013775291	1
136	8	1	3.25	25.28	25.47	25.63	1.013844937	1
163	8	1	2.75	25.13	25.35	25.48	1.013927577	1
100	8.5	2	2.5	24.42	24.71	24.77	1.014332514	1
3	11	2	4.75	25.75	25.97	26.12	1.014368932	1
182	9	1	2.25	23.66	23.84	24	1.014370245	1
215	10	1	1.75	25.39	25.6	25.76	1.014572666	1
115	11	1	2	25.37	25.5	25.74	1.014584155	1
7	8	1	4	25.36	25.49	25.73	1.014589905	1
217	8	2	1.25	23.81	23.88	24.16	1.014699706	1
197	7.5	2	1.25	23.08	23.27	23.42	1.014731369	1

60	11	1	2	24.73	24.79	25.1	1.014961585	1
4	11	2	3.5	25.23	25.48	25.61	1.015061435	1
97	7	2	1.25	23.58	23.76	23.94	1.015267176	1
178	9	1	2.75	24.11	24.34	24.48	1.015346329	1
157	9	2	1.25	24.02	24.11	24.39	1.01540383	1
169	9	1	2.25	24.65	24.87	25.03	1.015415822	1
67	7	1	1	24.4	24.55	24.78	1.01557377	1
45	8	1	1.75	22.94	23.14	23.31	1.016129032	1
69	7	1	1.75	24.11	24.26	24.5	1.016175861	1
88	7	2	2.25	24.52	24.75	24.92	1.016313214	1
142	9	1	1.25	25.02	25.3	25.43	1.01638689	1
154	8	2	2	24.38	24.6	24.78	1.016406891	1
221	8	2	3	23.75	23.95	24.14	1.016421053	1
216	10	1	1.75	25.47	25.74	25.89	1.016489988	1
206	8	2	2.5	24.79	24.97	25.2	1.016538927	1
8	8	1	4	25.29	25.36	25.71	1.016607355	1
66	7	1	1.25	24.6	24.81	25.01	1.016666667	1
162	8	1	0.75	23.31	23.53	23.7	1.016731017	1
170	9	1	2.25	24.47	24.7	24.88	1.01675521	1
218	8	2	1.25	23.66	23.81	24.06	1.016906171	1
17	7	2	3.25	23.98	24.13	24.39	1.017097581	1
80	10	2	1.75	23.83	24.03	24.24	1.017205204	1
207	8	2	2.25	24.88	25.06	25.31	1.017282958	1
155	8	2	1.75	24.25	24.47	24.67	1.017319588	1
211	9	1	1	24.81	25.03	25.24	1.017331721	1
44	8	1	1.5	22.93	23.05	23.33	1.017444396	1
114	11	1	0.75	24.87	25.08	25.31	1.017691998	1
107	9	2	1.25	23.47	23.6	23.89	1.017895185	1
12	9	1	3.75	25.12	25.39	25.57	1.017914013	1
18	7	2	3.25	23.97	24.19	24.4	1.017939091	1
166	8.5	2	1.75	23.31	23.5	23.73	1.018018018	1
194	7.5	2	1.25	23.54	23.76	23.97	1.01826678	1
42	12	1	1.5	23.53	23.72	23.96	1.018274543	1
98	7	2	1	23.44	23.76	23.87	1.01834471	1
30	9	1	1.75	24.61	24.91	25.07	1.018691589	1
38	8	2	2.25	23.42	23.8	23.86	1.018787361	1
156	9	2	2	24.39	24.62	24.85	1.018860189	1
53	9	1	1.75	24.25	24.41	24.71	1.018969072	1
50	10	2	1.5	24.19	24.42	24.65	1.019016122	1
56	8	1	1.75	24.56	24.68	25.03	1.019136808	1
165	8.5	2	1.5	23.46	23.76	23.91	1.019181586	1
49	7	2	5	24.42	24.77	24.89	1.019246519	1
164	8	1	3	25.02	25.27	25.51	1.019584333	1
59	10	2	5	25.05	25.22	25.55	1.01996008	1
72	8	1	2	24.87	25.14	25.37	1.020104544	1
68	7	1	1.75	23.98	24.07	24.47	1.020433695	1
57	8	1	2.5	24.68	24.79	25.19	1.020664506	1
73	8	1	2	24.59	24.85	25.1	1.020740138	1
29	9	1	2	24.66	24.96	25.18	1.02108678	1
102	9	1	1.5	24.6	24.87	25.12	1.021138211	1
101	9	1	0.75	24.49	24.78	25.01	1.021233156	1

75	8.5	1	1.75	24.45	24.85	24.97	1.021267894	1
143	9	1	1	24.87	25.16	25.4	1.021310816	1
11	9	1	3.5	25.15	25.5	25.69	1.021471173	1
82	8	2	3.75	24.88	25.18	25.42	1.02170418	1
140	11.5	1	1.25	24.73	24.97	25.27	1.021835827	1
52	9	1	2.5	24.37	24.58	24.92	1.022568732	1
24	7	2	4.75	25.3	25.65	25.88	1.022924901	1
81	8	2	3.5	24.83	25.17	25.41	1.02335884	1
22	9	2	0.75	23.38	23.69	23.93	1.02352438	1
199	10	2	1	23.76	24.1	24.33	1.023989899	1
32	8	2	2.5	24.17	24.64	24.75	1.02399669	1
198	10	2	1.75	24.38	24.71	24.98	1.024610336	1
23	7	2	4.25	25.11	25.45	25.74	1.025089606	1
85	9.5	2	2	23.39	23.59	23.99	1.025651988	1
79	10	2	1.5	23.68	24.03	24.29	1.025760135	1
86	9.5	2	1.75	23.43	23.66	24.07	1.027315408	1
48	7	2	4	24.41	24.88	25.11	1.028676772	1
141	11.5	1	1	24.58	24.91	25.3	1.029292107	1
71	7.5	2	1.75	24.3	24.47	25.09	1.032510288	1
87	7	2	1.75	24.17	24.83	24.98	1.033512619	1
70	7.5	2	1.75	24.22	24.46	25.05	1.034269199	1
74	8.5	1	2.5	24.59	25.16	25.45	1.034973566	1
219	10	2	1	24.39	24.52	25.54	1.047150472	1
220	10	2	0.75	24.15	24.31	25.64	1.061697723	1

CCT	AD	LT	WTW	Photopic pupil diameter	Mesopic pupil diameter	K1 Flat	K2 Steep	AST
572	3.61	3.22	13.04	2.79	5.61	42.13	44.7	2.57
508	3.32	3.27	12.33	4.05	6.86	43.6	44.47	0.87
546	3.1	3.64	11.32	4.92	7.02	44.29	45.18	0.89
559	3.48	3.31	12.29	5.18	7.44	43.83	45.12	1.29
572	3.6	3.2	12.87	2.89	6.57	41.77	44.53	2.76
524	3.36	3.42	12.02	4.67	6.58	42.88	44.88	2.00
525	3.4	3.3	12.09	4.25	6.27	42.51	43.27	0.76
528	2.82	3.77	11.25	3.18	5.28	45.86	46.68	0.82
556	3.26	3.33	12.05	5.28	7.26	42.35	44.06	1.71
533	3.17	3.25	11.65	5.92	7.54	45.18	46.68	1.5
523	3.33	3.38	12.04	4.91	6.9	43.05	44.58	1.53
532	3.48	3.42	12.34	5.29	6.77	44.12	45.79	1.67
527	3.34	3.29	12.25	3.9	5.9	42.35	43.27	0.92
519	3.32	3.29	12.44	3.63	6.78	43.66	44.94	1.28
547	3.29	3.32	11.91	5.13	6.9	42.40	44.23	1.83
573	3.2	3.45	11.84	5.2	7.02	42.78	43.83	1.05
504	3.27	3.37	12.18	6.45	8.08	41.41	42.29	0.88
573	3.19	3.43	11.88	4.31	6.11	43.38	45.12	1.74
544	3.41	3.51	12.55	3.48	6.45	41.56	43.44	1.88
587	3.27	3.09	12.21	3.63	6.65	41.36	42.94	1.58
541	3.21	3.3	11.81	3.15	6.42	44.12	45.06	0.94
533	3.11	3.44	11.41	4.73	7.47	46.3	46.87	0.57
543	3.28	3	11.83	3.2	6.43	43.55	45.61	2.06
509	3.24	3.37	12.27	5.89	7.8	41.36	42.19	0.83
593	3.16	3.26	11.79	4.68	6.37	42.35	43.55	1.20
584	3.23	3.19	12.35	3.13	6.5	41.31	42.94	1.63
598	3.26	3.23	12.01	5.78	7.39	42.45	44	1.55
581	3.38	3.19	12.16	3.82	7.14	42.4	44.41	2.01
517	3.11	3.42	12.01	4.4	6.32	42.61	43.83	1.22
580	3.35	3.52	11.66	4.86	7.19	41.67	42.72	1.06
525	3.28	3.28	12.23	3.53	6.6	44.82	45.86	1.04
547	2.8	3.59	11.76	5.38	6.83	42.88	43.27	0.39
607	3.27	3.42	12.12	3.27	6.31	41.21	42.03	0.82
509	2.96	3.38	11.56	4.77	6.33	44.82	45.24	0.42
551	3.41	3.2	12.16	3.86	6.04	41.62	43.55	1.93
544	3.04	3.37	12.24	3.3	6.07	41.87	43.83	1.96
511	2.97	3.35	11.47	3.67	6.17	45.3	46.17	0.87
511	3.33	3.23	12.26	5.56	7.7	42.67	43.89	1.22
563	2.97	3.42	11.48	3.63	5.51	44	44.58	0.58
505	3.73	3.42	12.87	3.95	7.68	43.16	44.53	1.37
530	2.95	3.36	11.65	3.54	5.81	42.67	43.16	0.49
580	3.28	3.65	13.64	3.53	6.74	40.96	42.67	1.71
556	3.2	3.36	12.49	5.03	7.57	42.83	43.83	1
528	3.43	3.24	12.18	3.85	5.58	44.23	45.3	1.07
537	3.48	3.45	12.21	6.5	8.16	44.53	46.23	1.70
508	3.41	3.42	12.16	4.18	5.85	43.16	44.7	1.54
540	3.36	3.02	12.07	4.61	6.25	43.21	45.42	2.21
540	3.4	3.41	12.32	4.3	6.39	41.82	43.86	1.84

552	3.09	3.65	11.42	4.46	6.52	44	45.3	1.3
578	3.23	0.39	12.07	4.89	6.71	42.24	43.21	0.97
545	2.82	3.53	11.82	5.24	6.9	42.94	43.38	0.44
506	3.75	3.39	12.59	5.77	7.98	43.83	44.94	1.11
574	3.27	3.58	12.37	2.96	5.98	42.78	43.95	1.17
526	3.49	3.25	12.32	4.08	6.15	44.58	46.04	1.46
515	3.13	3.51	11.96	3.36	5.68	44.23	46.62	2.39
582	3.29	3.17	11.99	4.29	5.93	42.67	43.55	0.88
558	3.49	3.32	12.14	5.52	7.49	43.95	45.18	1.23
528	2.83	3.7	11.34	3.54	5.74	45.67	47.14	1.47
597	3.26	3.55	12.68	2.84	6.91	39.02	39.66	0.64
573	2.94	3.34	11.45	3.02	5.19	43.44	44.64	1.2
508	3.81	2.99	12.29	3.63	6.52	43.38	44.64	1.26
565	2.94	3.37	11.64	3.99	8.74	43.1	43.95	0.85
533	3.35	3.12	12.07	4.63	6.98	41.46	42.99	1.53
554	2.94	3.11	11.89	4.26	6.18	42.51	44.35	1.84
588	3.36	3.54	11.99	5.38	7.87	41.56	42.29	0.73
498	3.66	3.31	12.3	4.07	8.07	44.53	44.86	0.35
551	3.06	3.35	12.33	3.67	6.92	41.46	43.83	2.37
543	3.29	3.75	11.73	4.73	8.2	42.88	44.94	2.06
534	3.38	3.48	12.36	5.19	6.87	41.67	43.05	1.38
522	3.42	3.42	12.15	5.46	7.02	42.67	43.83	1.16
525	3.4	3.45	12.27	5.06	6.77	42.94	44.23	1.29
558	3.1	3.47	11.85	3.18	6.42	42.35	42.78	0.67
575	3.25	3.28	11.83	4.81	6.97	42.61	43.6	0.99
550	3.45	3.2	11.4	3.87	6.26	43.49	44.53	1.04
589	3.15	3.25	11.89	5.43	6.9	42.03	43.77	1.74
570	3.24	3.35	12.09	4.31	6.14	42.19	43.32	1.13
516	3.19	3.34	11.88	3.29	5.89	44.41	47.14	2.73
505	2.99	3.28	12.19	3.28	5.97	40.52	40.71	0.19
589	3.42	3.47	12.03	5.66	7.08	42.4	42.99	0.59
560	3.24	3.58	12.14	3.26	6.08	43.27	44.23	0.96
531	2.86	3.42	11.72	3.23	5.77	43.89	44.47	0.58
533	3.31	3.22	11.99	5.27	6.49	43.05	44.35	1.3
593	3.37	3.43	12.01	5.6	7.21	42.13	42.83	0.7
562	3.11	3.69	12.21	4.44	7.89	43.44	44.29	0.85
541	3.02	3.44	11.4	3.87	7.35	46.23	47.14	0.91
566	3.62	3.42	13.21	3.85	5.94	43.38	44.76	1.38
542	3.31	3.77	11.78	7.49	8.45	43.16	44.58	1.42
523	3.27	3.32	12.75	3.36	6.61	44.12	46.04	1.92
532	3.36	3.46	12.34	4.85	6.7	41.67	43.05	1.38
546	3.4	3.4	12.48	4.9	7.11	43.83	46.04	2.21
555	3.22	3.34	12.02	3.87	7.53	42.94	43.16	0.22
506	2.98	3.31	11.93	3.89	6.37	41.36	41.62	0.26
543	3.29	3.55	11.95	3.54	6.01	43.44	44.47	1.03
603	3.06	3.41	12.1	3.27	5.9	42.78	44.41	1.63
512	3.4	3.47	12.15	4.21	6.27	43.60	44.58	0.98
527	3.04	3.53	11.81	3.31	6.31	43.05	44.12	1.07
561	3.13	3.44	11.72	3.23	6.11	42.51	42.61	0.1
548	3.41	3.2	11.31	3.52	6.11	43.49	44.12	0.63
531	3.1	3.66	12.69	3.68	7.05	43.32	44.41	1.09

511	3.44	3.4	12.29	5.24	6.67	43.49	45.24	1.75
552	2.96	3.09	11.82	4.63	6.93	43.1	44.47	1.37
569	3.08	3.28	11.43	3.07	5.7	44.35	46.75	2.40
553	3.04	3.32	12.19	5.65	7.11	42.83	44.35	1.52
568	3.31	3.49	12.36	3	6.02	42.61	44	1.39
571	3.35	3.31	12.3	3.24	6.55	41.41	42.35	0.94
578	3.48	3.51	12.3	5.34	6.61	42.03	42.61	0.58
568	3.63	3.41	12.87	3.74	6.49	43.43	44.64	1.32
545	3.24	3.39	11.67	5.61	6.94	44.00	44.70	0.70
519	3.05	3.57	12.26	4.72	6.42	42.51	43.55	1.04
515	3.3	3.4	11.91	3.66	7.35	44.47	46.11	1.64
605	3.22	3.07	11.54	3.45	6.29	42.72	43.60	0.88
627	3.14	3.09	11.69	3.42	5.66	42.24	42.83	0.59
572	3.04	3.37	12.3	4.37	7.43	41.51	42.61	1.1
556	2.87	3.23	11.93	2.81	6.12	42.88	43.89	1.01
574	2.63	3.62	11.99	6.57	6.68	41.51	43.21	1.7
572	3.11	3.75	12.31	4.3	7.7	44.41	45.3	0.89
608	3.31	3.39	12.26	3.48	6.68	41.31	42.13	0.82
571	2.83	3.74	11.48	4.31	6.41	42.29	44.06	1.77
564	3.26	3.38	11.73	3.45	5.46	42.99	45.06	2.07
569	3.31	3.32	12.36	4.54	6.64	41.31	42.19	0.88
594	3.55	3.44	12.64	5.75	7.22	42.78	44	1.22
536	2.86	3.41	11.31	3.15	5.64	43.77	44.23	0.46
539	3.39	3.53	12.56	3.52	6.42	42.08	42.61	0.53
582	3.57	3.45	12.65	5.86	7.35	42.99	44.53	1.54
567	3.15	3.29	11.36	2.86	5.2	44.53	46.87	2.34
611	3.35	3.26	11.57	3.6	6.03	43.21	45.73	2.52
594	2.92	3.47	12.05	3.37	5.79	43.21	43.32	0.11
518	3.02	3.34	11.95	4.29	6.16	43.21	45.55	2.34
577	3.52	3.43	12.44	5.02	6.45	41.93	42.72	0.79
570	3.07	3.36	12.14	4.87	8.31	41.41	42.67	1.26
542	2.92	3.51	12.77	3.85	6.56	43.49	45.98	2.49
526	3.46	3.4	12.6	3.53	6.71	42.94	44.41	1.47
575	2.87	2.96	11.49	3.44	6.2	44.58	45.61	1.03
566	3.08	3.53	12.11	6.62	7.83	41.21	41.31	0.1
524	2.97	3.41	11.71	3.69	5.74	42.29	43.21	0.92
572	3.57	3.55	11.63	3.69	6.21	44.64	46.68	2.04
624	3.32	3.23	11.62	3.9	6.55	43.10	45.92	2.82
514	3.03	3.53	11.83	3.16	6.47	43.1	44.06	0.96
557	3.27	3.34	11.79	3.2	6.17	43.32	44.76	1.44
564	3.17	3.42	11.52	4.29	6.03	44.12	45.12	1
505	3.67	2.87	12.18	5.05	6.86	43.55	44.41	0.86
561	3.27	3.39	12.1	5.48	7.21	42.19	44.29	2.1
578	2.77	3.87	11.57	4.63	6.47	42.03	44.23	2.20
603	3.04	3.51	11.62	4.07	6.98	41.46	41.62	0.16
600	2.92	3.51	11.65	3.19	5.57	43.21	43.38	0.17
481	3.79	3.13	12.08	4.92	6.96	41.93	42.94	1.01
508	3.11	3.37	12.44	3.8	7.11	41.11	41.7	0.56
550	3.37	3.38	12.1	3.69	5.64	43.10	44.23	1.13
563	3.11	3.48	12.25	3.26	6.53	42.08	42.67	0.59
524	3.26	3.42	12.04	3.82	7.21	44.23	46.23	2

569	3.28	3.23	12.1	4.03	6.69	42.51	43.55	1.04
610	2.98	3.54	11.62	4.01	7.09	41.26	41.87	0.61
521	3.33	3.32	11.99	3.7	6.37	43.77	46.87	3.10
507	3.03	3.59	11.96	4.14	6.23	42.29	43.68	1.37
548	3.09	3.5	12.3	4.32	5.37	41.01	42.03	1.02
602	3.27	3.17	11.5	4.49	6.14	42.4	43.43	0.92
559	3.49	3.46	12.36	3.34	5.42	41.51	42.19	0.68
574	3.1	3.37	11.54	3.27	5.24	45.06	45.86	0.80
554	3.15	3.63	11.98	4.06	6.69	41.93	42.78	0.85
586	3.07	3.3	12.05	3.65	7.22	42.03	42.67	0.64
626	3.31	3.29	12.29	5.26	7.14	41.72	42.51	0.79
495	3.41	3.19	12.42	4.15	7.33	42.4	43.44	1.04
525	3.05	3.67	12.15	3.34	7.02	43.38	44.29	0.91
474	3.82	3.11	12.03	4.67	6.73	42.13	43.21	1.08
532	3.29	3.07	11.95	4.57	5.51	42.61	44.29	1.68
559	3.32	3.39	11.91	3.83	5.67	42.72	43.49	0.77
556	3.21	3.25	12.79	4.6	6.12	41.46	41.77	0.31
554	3.16	3.59	11.95	3.89	7.62	43.27	43.55	0.28
608	3.27	3.2	11.38	3.23	5.85	42.61	43.83	1.22
553	3.06	3.51	12.4	3.36	5.77	42.51	43.16	0.65
557	3.18	3.47	11.57	3.82	5.37	44.47	45.49	1.02
580	3.29	3.41	11.25	7.66	8	43.83	45.06	1.23
523	3.32	3.05	12.14	3.99	5.64	42.45	43.95	1.5
495	3.41	3.21	12.26	4.07	7.17	42.19	43.1	0.91
554	3.13	3.55	12.31	6.64	7.94	41.11	41.41	0.3
570	3.09	3.37	11.71	3.56	5.66	45.24	46.11	0.87
507	3.1	3.34	12.13	5.75	7.29	41.16	41.93	0.77
575	2.65	3.55	11.81	6.82	7.22	41.87	43.49	1.62
534	3.38	3.33	12.05	3.72	6.79	43.27	45.12	1.85
559	3.25	3.32	11.61	3.59	5.84	44.12	45.12	1.00
545	2.96	3.34	11.56	3.54	4.63	43.05	44.47	1.42
542	3.06	3.5	12.05	3.45	7.02	43.16	44.58	1.42
540	3.43	3.4	12.21	5.02	7.05	44.23	46.04	1.81
522	3.36	3.36	11.87	3.39	6.61	44	47.01	3.01
514	3.26	3.6	12.48	4.02	7.19	43.89	44.29	0.40
512	2.92	3.44	11.87	4.71	6.32	43.66	45.98	2.32
553	3.18	3.47	12.25	3.99	5.88	41.67	42.83	1.16
546	3.2	3.35	12.15	6.29	7.56	43.21	44.35	1.14
522	3.4	3.4	12.62	3.59	6.44	42.72	44.23	1.51
529	3.03	3.61	12.18	4.46	7.41	41.82	42.08	0.26
549	2.98	3.3	11.64	3.74	4.64	42.94	44.12	1.18
494	3.4	3.64	12.32	5.4	7.01	41.36	42.61	1.25
569	3.26	3.38	12.04	4.65	7.16	42.45	43.95	1.5
557	3.02	3.33	12.05	3.88	7.12	42.56	44.12	1.56
516	3.38	3.24	12.39	5.9	7.63	42.08	43.44	1.36
548	3.12	3.64	12.11	3.56	7.66	42.13	42.56	0.43
528	2.99	3.67	12.21	3.59	6.83	41.62	41.82	0.20
526	3.4	3.23	12.49	5.88	7.55	42.35	43.72	1.37
518	3.46	3.36	12.48	4.85	6.89	43.55	44.06	0.51
577	3.42	3.34	12.72	5.76	7.42	41.56	43.32	1.76
577	3.39	3.34	12.72	6.02	8.04	41.31	43.32	2.01

616	3.16	3.32	12.05	3.28	5.08	41.62	43.66	2.04
627	3.33	3.26	12.33	3.62	7.23	41.77	42.67	0.9
519	3.56	3.24	12.18	4.29	7.28	44.06	46.11	2.05
536	3.09	3.52	12.38	3.66	6.63	41.56	43.49	1.93
555	3.63	2.92	13.43	3.04	5.87	42.45	44	1.55
552	3.27	3.37	11.98	5.02	7.5	43.16	44.12	0.96
570	3.12	3.26	12.16	3.64	6.82	42.78	44.53	1.75
533	3.06	3.53	12.52	5.13	6.52	41.72	43.32	1.96
550	3.25	3.39	11.88	5.1	6.11	43.60	44.64	1.04
519	3.08	3.2	11.91	3.48	6.11	43.38	45.12	1.74
515	3.36	3.48	12.02	4.78	6.56	43.32	43.77	0.45
510	3.08	3.17	12.05	5.2	7.02	42.78	43.83	1.05
570	3.12	3.17	11.56	3.53	6.35	42.51	43.89	1.38
485	2.91	3.69	12	4.48	6.89	43.95	44.64	0.69
573	3.05	3.41	11.35	3.57	7.03	44.12	45.06	0.94
474	2.93	3.68	11.73	3.88	6.4	43.55	44.18	0.63
497	3.44	3.62	12.44	5.15	6.82	41.46	42.72	1.26
563	3.6	2.95	12.94	3.28	5.77	42.45	44	1.55
512	3.24	3.32	12.5	3.65	6.19	41.82	42.88	1.06
530	3.2	3.33	12.08	4.91	7.47	42.19	43.6	1.41
506	3.26	3.3	12.42	4.99	6.79	42.51	43.6	1.09
612	3.14	3.33	12.46	5.11	7.97	41.67	43.55	1.88
565	3.72	3.14	12.1	5.04	6.66	43.21	44.06	0.85
559	3.68	3.11	12.17	3.87	6.6	43.38	44.58	1.2

T.Sph（M）	T.Sph（6mm）	T.Sph（4mm）	T.Coma（M）	T.Coma（6mm）	T.Coma（4mm）	T.Tre（M）	T.Tre（6mm）	T.Tre（4mm）
0.067	0.11	0.009	0.12	0.169	0.035	0.202	0.212	0.133
0.068	0.067	0.013	0.098	0.118	0.033	0.288	0.255	0.11
0.32	0.244	0.064	0.508	0.342	0.098	0.379	0.288	0.082
0.302	0.19	0.014	0.303	0.216	0.036	0.44	0.226	0.083
0.023	0.026	0.009	0.235	0.175	0.048	0.233	0.201	0.08
0.165	0.145	0.037	0.166	0.116	0.044	0.313	0.278	0.126
0.196	0.183	0.065	0.101	0.094	0.043	0.318	0.282	0.1
0.042	0.067	0.003	0.278	0.298	0.141	0.083	0.092	0.049
0.079	0.084	0.011	0.441	0.245	0.05	0.321	0.241	0.088
0.077	0.014	0.01	0.369	0.153	0.056	0.122	0.087	0.095
0.142	0.102	0.008	0.195	0.144	0.046	0.198	0.135	0.043
0.246	0.146	0.028	0.324	0.192	0.045	0.436	0.364	0.158
0.216	0.226	0.058	0.223	0.243	0.042	0.231	0.242	0.081
0.097	0.068	0.038	0.41	0.219	0.032	0.939	0.625	0.23
0.165	0.165	0.04	0.497	0.332	0.053	0.285	0.244	0.105
0.3	0.111	0.023	0.434	0.092	0.033	0.317	0.239	0.083
0.083	0.061	0.013	0.208	0.155	0.057	0.56	0.335	0.154
0.255	0.238	0.034	0.103	0.088	0.015	0.365	0.344	0.175
0.033	0.035	0.02	0.216	0.207	0.104	0.437	0.324	0.129
0.2	0.148	0.041	0.13	0.105	0.028	0.206	0.152	0.067
0.15	0.124	0.027	0.114	0.105	0.038	0.131	0.125	0.054
0.321	0.222	0.043	0.519	0.329	0.068	0.551	0.319	0.141
0.079	0.048	0.029	0.359	0.26	0.053	0.227	0.206	0.079
0.048	0.03	0.004	0.357	0.223	0.078	0.603	0.363	0.157
0.144	0.118	0.016	0.254	0.184	0.037	0.366	0.349	0.149
0.234	0.17	0.016	0.213	0.125	0.046	0.306	0.145	0.072
0.443	0.244	0.05	0.255	0.108	0.007	0.373	0.225	0.072
0.118	0.106	0.46	0.275	0.145	0.051	0.237	0.181	0.094
0.351	0.28	0.024	0.194	0.17	0.093	0.247	0.242	0.115
0.123	0.107	0.011	0.636	0.396	0.106	0.53	0.354	0.138
0.115	0.097	0.031	0.197	0.096	0.028	0.181	0.114	0.016
0.188	0.136	0.037	0.236	0.147	0.034	0.137	0.116	0.087
0.022	0.015	0.013	0.276	0.241	0.08	0.102	0.095	0.04
0.062	0.059	0.023	0.24	0.212	0.072	0.284	0.258	0.125
0.07	0.067	0.004	0.425	0.42	0.13	0.318	0.315	0.093
0.195	0.176	0.018	0.244	0.209	0.055	0.284	0.284	0.203
0.078	0.074	0.021	0.06	0.057	0.025	0.236	0.227	0.115
0.034	0.024	0.013	0.45	0.22	0.067	0.744	0.47	0.151
0.065	0.083	0.027	0.126	0.155	0.051	0.101	0.104	0.075
0.19	0.047	0	0.076	0.072	0.03	0.253	0.197	0.064
0.149	0.199	0.024	0.147	0.181	0.079	0.337	0.37	0.16
0.088	0.08	0.044	0.184	0.175	0.067	0.384	0.339	0.156
0.181	0.138	0.024	0.321	0.145	0.047	0.226	0.152	0.035
0.058	0.071	0.006	0.202	0.215	0.105	0.247	0.244	0.183
0.23	0.139	0.024	0.307	0.147	0.04	0.737	0.338	0.126
0.04	0.043	0.012	0.213	0.236	0.054	0.042	0.037	0.011
0.047	0.049	0.017	0.244	0.199	0.096	0.346	0.3	0.091
0.111	0.065	0.008	0.278	0.233	0.15	0.06	0.05	0.02

0.228	0.21	0.065	0.31	0.247	0.083	0.589	0.49	0.225
0.014	0.021	0.032	0.079	0.071	0.038	0.192	0.137	0.017
0.147	0.139	0.023	0.1	0.093	0.023	0.102	0.028	0.016
0.223	0.27	0.009	0.389	0.129	0.034	0.234	0.126	0.035
0.156	0.159	0.043	0.13	0.133	0.046	0.432	0.431	0.274
0.06	0.054	0.003	0.145	0.118	0.048	0.312	0.268	0.064
0.052	0.078	0.016	0.058	0.092	0.026	0.129	0.168	0.055
0.121	0.125	0.032	0.033	0.037	0.016	0.186	0.184	0.118
0.36	0.204	0.043	0.341	0.183	0.05	0.554	0.34	0.111
0.084	0.086	0.022	0.208	0.234	0.059	0.409	0.447	0.13
0.266	0.154	0.005	0.263	0.15	0.034	0.622	0.416	0.196
0.045	0.045	0.042	0.085	0.099	0.035	0.059	0.06	0.046
0.073	0.078	0.047	0.452	0.365	0.09	0.356	0.323	0.173
0.521	0.051	0.007	0.535	0.165	0.032	0.912	0.224	0.095
0.098	0.103	0.033	0.26	0.158	0.031	0.168	0.159	0.09
0.148	0.152	0.088	0.184	0.135	0.052	0.188	0.164	0.033
0.199	0.078	0.024	0.479	0.161	0.044	0.334	0.213	0.121
0.347	0.349	0.068	0.606	0.299	0.064	0.406	0.076	0.022
0.71	0.4	0.043	0.743	0.482	0.088	0.352	0.312	0.132
0.261	0.188	0.043	0.384	0.197	0.058	0.686	0.347	0.106
0.175	0.175	0.059	0.153	0.114	0.043	0.15	0.14	0.084
0.139	0.038	0.014	0.181	0.142	0.028	0.465	0.376	0.184
0.082	0.036	0.012	0.163	0.138	0.056	0.22	0.186	0.11
0.099	0.098	0.026	0.666	0.597	0.204	0.498	0.469	0.211
0.089	0.017	0.006	0.273	0.213	0.054	0.421	0.25	0.072
0.093	0.076	0.01	0.147	0.104	0.048	0.311	0.292	0.144
0.335	0.186	0.039	0.144	0.101	0.054	0.242	0.17	0.093
0.054	0.056	0.029	0.2	0.17	0.033	0.167	0.162	0.052
0.091	0.093	0.018	0.139	0.147	0.032	0.2	0.204	0.12
0.13	0.129	0.109	0.135	0.139	0.096	0.617	0.615	0.54
0.112	0.036	0.022	0.196	0.079	0.018	0.362	0.228	0.089
0.271	0.259	0.052	0.196	0.172	0.022	0.207	0.201	0.153
0.069	0.065	0.033	0.174	0.2	0.045	0.143	0.151	0.064
0.224	0.19	0.145	0.153	0.145	0.051	0.485	0.436	0.205
0.11	0.018	0.007	0.562	0.317	0.097	0.233	0.141	0.074
0.067	0.063	0.018	0.363	0.115	0.025	0.68	0.317	0.101
0.312	0.177	0.034	0.349	0.18	0.048	0.119	0.085	0.039
0.128	0.141	0.016	0.106	0.108	0.038	0.247	0.26	0.072
0.313	0.234	0.057	0.664	0.308	0.082	0.699	0.408	0.118
0.038	0.017	0.009	0.321	0.275	0.112	0.221	0.15	0.087
0.099	0.099	0.035	0.155	0.101	0.025	0.17	0.119	0.058
0.066	0.065	0.011	0.129	0.06	0.032	0.236	0.192	0.092
0.205	0.173	0.039	0.09	0.031	0.022	0.432	0.492	0.312
0.169	0.145	0.006	0.509	0.437	0.082	0.649	0.576	0.188
0.061	0.061	0.033	0.117	0.114	0.047	0.337	0.335	0.09
0.105	0.106	0.048	0.082	0.09	0.036	0.632	0.645	0.342
0.187	0.167	0.058	0.239	0.222	0.079	0.251	0.227	0.107
0.144	0.124	0.034	0.17	0.142	0.023	0.174	0.159	0.086
0.053	0.031	0.011	0.089	0.07	0.022	0.373	0.358	0.24
0.059	0.057	0.005	0.119	0.109	0.041	0.179	0.179	0.064
0.002	0.018	0.019	0.219	0.153	0.052	0.242	0.212	0.152

0.102	0.065	0.014	0.253	0.16	0.054	0.09	0.064	0.048
0.481	0.236	0.01	1.08	0.521	0.025	0.748	0.394	0.137
0.085	0.081	0.038	0.224	0.267	0.067	0.318	0.328	0.27
0.299	0.053	0.015	0.374	0.105	0.048	0.99	0.484	0.239
0.16	0.159	0.044	0.215	0.215	0.081	0.21	0.21	0.119
0.166	0.119	0.021	0.306	0.239	0.083	0.19	0.157	0.057
0.039	0.023	0.007	0.226	0.203	0.085	0.176	0.159	0.085
0.143	0.095	0.024	0.25	0.082	0.035	0.381	0.368	0.136
0.047	0.046	0.018	0.101	0.097	0.066	0.161	0.133	0.106
0.043	0.043	0.024	0.051	0.045	0.019	0.118	0.104	0.072
0.103	0.094	0.022	0.249	0.171	0.058	0.147	0.079	0.074
0.154	0.144	0.048	0.237	0.22	0.086	0.126	0.109	0.044
0.135	0.225	0.019	0.139	0.187	0.037	0.168	0.205	0.054
0.524	0.228	0.045	0.481	0.246	0.052	0.677	0.491	0.235
0.228	0.194	0.05	0.334	0.286	0.055	0.212	0.206	0.044
0.122	0.123	0.044	0.124	0.09	0.057	0.425	0.24	0.034
0.406	0.192	0.073	0.62	0.211	0.062	0.715	0.378	0.114
0.014	0.01	0.018	0.37	0.302	0.109	0.161	0.127	0.039
0.401	0.329	0.087	0.229	0.228	0.119	0.381	0.36	0.118
0.008	0.06	0.024	0.075	0.124	0.054	0.237	0.362	0.194
0.22	0.068	0.008	0.247	0.108	0.047	0.354	0.122	0.017
0.207	0.168	0.037	0.688	0.431	0.106	0.629	0.444	0.153
0.042	0.043	0.026	0.156	0.204	0.031	0.142	0.147	0.091
0.352	0.248	0.052	0.407	0.318	0.073	0.198	0.179	0.067
0.212	0.149	0.029	0.486	0.342	0.089	0.576	0.452	0.181
0.097	0.11	0.027	0.097	0.104	0.072	0.098	0.098	0.065
0.304	0.299	0.063	0.178	0.174	0.039	0.406	0.406	0.187
0.077	0.088	0.019	0.035	0.045	0.019	0.305	0.319	0.174
0.038	0.036	0.028	0.314	0.268	0.023	0.318	0.285	0.08
0.02	0.011	0.004	0.035	0.032	0.011	0.242	0.216	0.103
0.742	0.155	0.003	0.333	0.119	0.05	0.237	0.143	0.089
0.031	0.035	0.008	0.158	0.121	0.033	0.337	0.278	0.097
0.023	0.017	0.024	0.351	0.287	0.09	0.192	0.122	0.024
0.044	0.043	0.024	0.057	0.049	0.044	0.169	0.174	0.099
0.082	0.02	0.005	0.352	0.116	0.017	0.176	0.163	0.082
0.089	0.089	0.013	0.165	0.224	0.033	0.061	0.056	0.031
0.12	0.12	0.031	0.257	0.224	0.051	0.117	0.087	0.064
0.426	0.35	0.094	0.224	0.169	0.039	0.767	0.792	0.286
0.068	0.065	0.01	0.23	0.178	0.05	0.359	0.301	0.125
0.177	0.18	0.033	0.498	0.481	0.111	0.619	0.582	0.403
0.113	0.114	0.044	0.188	0.188	0.061	0.21	0.206	0.113
0.351	0.191	0.02	0.122	0.116	0.078	0.323	0.181	0.049
0.51	0.037	0.015	0.705	0.117	0.084	0.506	0.205	0.107
0.365	0.304	0.089	0.412	0.355	0.163	0.812	0.663	0.239
0.143	0.064	0.016	0.445	0.258	0.091	0.737	0.513	0.227
0.092	0.105	0.019	0.086	0.087	0.051	0.11	0.17	0.073
0.066	0.039	0.032	0.244	0.217	0.09	0.257	0.181	0.078
0.059	0.049	0.019	0.512	0.302	0.059	0.655	0.399	0.109
0.117	0.14	0.014	0.136	0.16	0.053	0.137	0.162	0.05
0.05	0.045	0.003	0.146	0.14	0.031	0.355	0.341	0.085
0.411	0.235	0.055	0.222	0.093	0.019	0.287	0.125	0.042

0.121	0.05	0.009	0.246	0.173	0.057	0.286	0.216	0.087
0.062	0.021	0.008	0.222	0.118	0.059	0.29	0.107	0.024
0.099	0.1	0.043	0.294	0.261	0.094	0.26	0.267	0.155
0.149	0.127	0.015	0.117	0.108	0.038	0.172	0.14	0.049
0.043	0.056	0.041	0.11	0.162	0.044	0.097	0.108	0.053
0.12	0.112	0.005	0.12	0.106	0.056	0.19	0.182	0.055
0.113	0.118	0.045	0.116	0.121	0.051	0.122	0.133	0.043
0.106	0.113	0.056	0.066	0.081	0.04	0.123	0.139	0.078
0.181	0.037	0.004	0.29	0.203	0.055	0.438	0.286	0.126
0.055	0.032	0.004	0.228	0.086	0.038	0.272	0.154	0.054
0.118	0.058	0.019	0.278	0.218	0.044	0.305	0.248	0.12
0.371	0.134	0.004	0.049	0.053	0.05	0.299	0.267	0.175
0.079	0.049	0.016	0.087	0.091	0.044	0.195	0.189	0.073
0.824	0.111	0.058	0.763	0.178	0.086	0.402	0.389	0.219
0.081	0.089	0.023	0.115	0.126	0.052	0.209	0.228	0.091
0.018	0.033	0.007	0.071	0.089	0.053	0.176	0.185	0.101
0.067	0.06	0.008	0.264	0.249	0.071	0.29	0.283	0.122
0.226	0.143	0.045	0.314	0.117	0.025	0.756	0.408	0.161
0.064	0.061	0.038	0.238	0.251	0.09	0.107	0.107	0.083
0.067	0.09	0.027	0.184	0.224	0.045	0.32	0.373	0.083
0.091	0.098	0.032	0.178	0.236	0.077	0.139	0.199	0.053
0.265	0.198	0.106	0.438	0.083	0.046	0.431	0.267	0.213
0.014	0.031	0.011	0.057	0.068	0.034	0.075	0.085	0.032
0.287	0.124	0.007	0.284	0.128	0.007	0.107	0.066	0.029
0.057	0.057	0.011	0.4	0.182	0.071	0.537	0.316	0.171
0.018	0.014	0.004	0.129	0.143	0.066	0.234	0.245	0.113
0.214	0.083	0.035	1.036	0.443	0.098	0.766	0.601	0.224
0.28	0.174	0.038	0.222	0.183	0.072	0.222	0.108	0.039
0.107	0.083	0.014	0.596	0.42	0.139	0.361	0.281	0.14
0.018	0.021	0.011	0.097	0.1	0.044	0.118	0.12	0.051
0.011	0.012	0.008	0.063	0.102	0.038	0.15	0.16	0.114
0.054	0.02	0.034	0.374	0.236	0.066	0.121	0.176	0.121
0.146	0.145	0.049	0.311	0.181	0.053	0.129	0.078	0.029
0.061	0.063	0.027	0.376	0.254	0.066	0.312	0.261	0.127
0.123	0.052	0.012	0.296	0.168	0.036	0.077	0.057	0.04
0.044	0.032	0.023	0.161	0.129	0.064	0.713	0.637	0.265
0.107	0.114	0.06	0.109	0.117	0.031	0.053	0.06	0.025
0.096	0.07	0.031	0.148	0.132	0.054	0.127	0.084	0.075
0.119	0.061	0.004	0.241	0.2	0.059	0.141	0.124	0.043
0.282	0.164	0.023	0.4	0.255	0.071	0.528	0.333	0.119
0.006	0.006	0.006	0.017	0.065	0.011	0.114	0.124	0.086
0.181	0.074	0.013	0.093	0.07	0.046	0.507	0.361	0.169
0.166	0.037	0.004	0.262	0.109	0.06	0.416	0.328	0.201
0.14	0.032	0.018	0.223	0.101	0.021	0.333	0.247	0.092
0.271	0.082	0.007	0.17	0.146	0.055	0.129	0.109	0.047
0.046	0.01	0.004	0.538	0.22	0.067	0.596	0.443	0.209
0.116	0.094	0.016	0.5	0.285	0.035	0.284	0.21	0.063
0.394	0.114	0.0004	0.343	0.209	0.101	0.37	0.276	0.145
0.054	0.024	0.014	0.139	0.135	0.059	0.11	0.058	0.014
0.053	0.048	0.017	0.218	0.143	0.062	0.285	0.147	0.045
0.107	0.031	0.004	0.485	0.108	0.019	0.603	0.3	0.117

0.023	0.023	0.018	0.131	0.142	0.069	0.103	0.107	0.075
0.125	0.03	0.011	0.237	0.169	0.069	0.364	0.179	0.041
0.04	0.033	0.026	0.471	0.271	0.095	0.4	0.215	0.094
0.206	0.153	0.049	0.248	0.171	0.014	0.526	0.438	0.197
0.206	0.221	0.088	0.26	0.294	0.077	0.153	0.152	0.064
0.111	0.115	0.037	0.074	0.085	0.051	0.177	0.185	0.112
0.086	0.049	0.004	0.413	0.277	0.035	0.497	0.338	0.078
0.169	0.141	0.046	0.597	0.488	0.164	0.131	0.078	0.032
0.06	0.063	0.033	0.21	0.194	0.064	0.416	0.412	0.186
0.255	0.238	0.034	0.103	0.088	0.015	0.365	0.344	0.175
0.064	0.056	0.021	0.235	0.19	0.061	0.224	0.187	0.098
0.3	0.111	0.023	0.434	0.092	0.033	0.317	0.239	0.083
0.1	0.08	0.035	0.27	0.236	0.038	0.246	0.188	0.096
0.211	0.14	0.008	0.286	0.189	0.048	0.579	0.351	0.135
0.464	0.245	0.029	0.189	0.108	0.021	0.105	0.033	0.038
0.223	0.176	0.038	0.178	0.112	0.083	0.525	0.435	0.173
0.048	0.017	0.006	0.16	0.105	0.023	0.149	0.141	0.1
0.122	0.123	0.037	0.067	0.068	0.018	0.127	0.128	0.062
0.147	0.125	0.017	0.103	0.101	0.052	0.473	0.443	0.165
0.104	0.113	0.031	0.354	0.044	0.026	0.144	0.048	0.021
0.224	0.139	0.013	0.215	0.172	0.077	0.142	0.122	0.071
0.271	0.127	0.042	1.309	0.33	0.059	0.783	0.805	0.488
0.421	0.349	0.07	0.192	0.12	0.029	0.199	0.117	0.02
0.312	0.261	0.057	0.204	0.169	0.067	0.295	0.247	0.112

T.HOA（M）	T.HOA（6mm）	T.HOA（4mm）	C.Sph（M）	C.Sph（6mm）	C.Sph（6mm）	C.Coma（M）	C.Coma（6mm）	C.Coma（4mm）
0.276	0.347	0.147	0.177	0.241	0.033	0.257	0.318	0.088
0.388	0.345	0.134	0.503	0.284	0.011	0.554	0.431	0.084
0.731	0.528	0.154	0.577	0.333	0.085	0.161	0.097	0.045
0.665	0.409	0.14	0.874	0.354	0.076	0.271	0.131	0.037
0.382	0.308	0.121	0.417	0.272	0.027	0.232	0.177	0.059
0.425	0.352	0.15	0.279	0.207	0.031	0.266	0.2	0.034
0.403	0.36	0.13	0.174	0.158	0.021	0.174	0.143	0.059
0.304	0.332	0.157	0.184	0.328	0.052	0.304	0.372	0.163
0.618	0.4	0.116	0.544	0.27	0.055	0.192	0.144	0.032
0.453	0.204	0.116	0.669	0.281	0.045	0.111	0.028	0.019
0.358	0.266	0.115	0.417	0.251	0.041	0.462	0.27	0.068
0.641	0.447	0.175	0.414	0.253	0.049	0.364	0.257	0.073
0.418	0.448	0.115	0.282	0.295	0.072	0.374	0.391	0.089
1.098	0.719	0.257	0.543	0.37	0.083	0.604	0.433	0.136
0.641	0.491	0.146	0.525	0.335	0.052	0.3	0.222	0.018
0.949	0.311	0.103	0.547	0.296	0.057	0.154	0.067	0.017
0.677	0.408	0.179	0.52	0.19	0.047	0.682	0.169	0.034
0.522	0.488	0.196	0.325	0.305	0.061	0.159	0.152	0.052
0.529	0.405	0.172	0.424	0.359	0.063	0.11	0.126	0.046
0.364	0.266	0.104	0.321	0.205	0.021	0.139	0.123	0.074
0.271	0.233	0.088	0.356	0.276	0.059	0.255	0.189	0.032
0.932	0.531	0.171	1.144	0.474	0.105	0.295	0.152	0.057
0.449	0.35	0.111	0.272	0.215	0.047	0.204	0.15	0.029
0.729	0.439	0.185	0.498	0.182	0.035	0.592	0.219	0.086
0.559	0.491	0.184	0.08	0.047	0.004	0.576	0.445	0.083
0.636	0.4	0.166	0.335	0.235	0.047	0.189	0.167	0.065
0.684	0.393	0.104	0.57	0.26	0.048	0.273	0.142	0.054
0.52	0.285	0.129	0.527	0.271	0.063	0.271	0.107	0.041
0.531	0.442	0.159	0.148	0.104	0.021	0.673	0.554	0.148
0.846	0.551	0.178	0.527	0.25	0.041	0.351	0.221	0.079
0.481	0.307	0.091	0.407	0.265	0.034	0.231	0.144	0.052
0.421	0.288	0.12	0.304	0.214	0.046	0.328	0.21	0.073
0.337	0.297	0.105	0.352	0.28	0.033	0.081	0.058	0.029
0.384	0.346	0.155	0.321	0.252	0.049	0.153	0.129	0.037
0.526	0.555	0.187	0.321	0.32	0.08	0.239	0.226	0.072
0.541	0.506	0.271	0.724	0.671	0.109	0.309	0.292	0.119
0.28	0.265	0.125	0.245	0.218	0.051	0.164	0.146	0.029
0.909	0.542	0.191	0.708	0.268	0.059	0.385	0.127	0.026
0.211	0.273	0.103	0.071	0.1	0.013	0.263	0.356	0.094
0.353	0.237	0.093	0.484	0.2	0.051	0.59	0.258	0.085
0.417	0.486	0.184	0.129	0.138	0.045	0.142	0.16	0.064
0.471	0.41	0.182	0.358	0.251	0.084	0.045	0.049	0.034
0.47	0.284	0.075	0.622	0.287	0.047	0.849	0.321	0.102
0.366	0.402	0.224	0.151	0.148	0.068	0.095	0.282	0.073
0.889	0.412	0.145	0.93	0.264	0.05	0.238	0.108	0.038
0.263	0.287	0.104	0.151	0.169	0.029	0.472	0.513	0.132
0.492	0.418	0.14	0.207	0.186	0.034	0.318	0.283	0.08
0.41	0.35	0.171	0.245	0.214	0.049	0.632	0.51	0.198

0.725	0.604	0.257	0.39	0.293	0.077	0.303	0.263	0.147
0.286	0.214	0.079	0.341	0.229	0.044	0.193	0.1	0.018
0.252	0.199	0.053	0.233	0.143	0.012	0.22	0.133	0.048
0.563	0.23	0.079	0.425	0.156	0.042	0.7	0.204	0.059
0.497	0.498	0.301	0.336	0.342	0.09	0.16	0.16	0.113
0.62	0.578	0.494	0.46	0.403	0.033	0.508	0.444	0.056
0.195	0.289	0.083	0.211	0.262	0.053	0.205	0.248	0.049
0.247	0.252	0.136	0.277	0.297	0.054	0.283	0.302	0.086
0.776	0.45	0.138	0.755	0.284	0.04	0.263	0.107	0.025
0.578	0.62	0.236	0.203	0.244	0.081	0.34	0.386	0.064
0.817	0.548	0.236	0.168	0.104	0.023	0.394	0.205	0.025
0.192	0.203	0.138	0.077	0.122	0.037	0.225	0.408	0.084
0.629	0.538	0.215	0.385	0.29	0.046	0.124	0.12	0.065
1.264	0.293	0.104	0.7	0.242	0.068	0.638	0.107	0.01
0.373	0.279	0.119	0.924	0.458	0.112	1.354	0.595	0.111
0.407	0.359	0.131	0.767	0.678	0.07	0.596	0.554	0.037
0.648	0.29	0.136	0.728	0.261	0.051	0.289	0.156	0.056
0.855	0.48	0.113	0.948	0.571	0.087	1.102	0.475	0.12
1.16	0.746	0.193	1.183	0.712	0.118	0.441	0.21	0.08
0.918	0.49	0.145	0.586	0.272	0.095	1.062	0.304	0.059
0.335	0.28	0.121	0.439	0.244	0.032	0.059	0.05	0.02
0.538	0.413	0.189	0.246	0.141	0.022	0.212	0.092	0.017
1.224	1.017	0.614	0.276	0.191	0.052	0.392	0.237	0.054
0.895	0.819	0.36	0.195	0.135	0.029	0.769	0.678	0.271
0.63	0.365	0.102	0.326	0.183	0.037	0.382	0.141	0.021
0.368	0.329	0.155	0.213	0.183	0.048	0.124	0.109	0.033
0.492	0.308	0.135	0.17	0.072	0.01	0.474	0.228	0.043
0.347	0.315	0.096	0.221	0.201	0.021	0.323	0.296	0.066
0.31	0.325	0.139	0.255	0.276	0.041	0.227	0.242	0.026
0.815	0.815	0.636	0.232	0.239	0.036	0.622	0.625	0.335
0.486	0.269	0.097	0.182	0.135	0.053	0.58	0.311	0.07
0.428	0.404	0.179	0.174	0.161	0.091	0.25	0.242	0.106
0.258	0.291	0.092	0.21	0.24	0.055	0.239	0.278	0.067
1.045	0.902	0.46	0.24	0.201	0.062	0.231	0.187	0.078
0.67	0.365	0.126	0.196	0.117	0.026	0.661	0.35	0.115
0.825	0.365	0.128	0.401	0.121	0.015	1.34	0.497	0.136
0.56	0.3	0.092	1.007	0.443	0.095	0.367	0.207	0.065
0.401	0.421	0.135	0.346	0.361	0.07	0.019	0.019	0.018
1.055	0.57	0.165	0.483	0.22	0.064	1.605	0.567	0.14
0.605	0.452	0.166	0.352	0.258	0.054	0.249	0.212	0.059
0.29	0.23	0.104	0.27	0.183	0.039	0.201	0.126	0.05
0.345	0.263	0.123	0.383	0.212	0.045	0.809	0.444	0.107
0.669	0.553	0.337	0.707	0.307	51	0.595	0.27	0.067
0.874	0.769	0.261	0.467	0.254	0.119	1.163	0.501	0.098
0.395	0.391	0.118	0.258	0.255	0.046	0.23	0.229	0.051
0.67	0.685	0.363	0.266	0.287	0.055	0.194	0.208	0.051
0.493	0.432	0.163	0.297	0.264	0.073	0.221	0.19	0.039
0.328	0.281	0.102	0.152	0.129	0.031	0.518	0.442	0.122
0.412	0.392	0.28	0.241	0.219	0.017	0.247	0.23	0.061
0.241	0.228	0.079	0.179	0.167	0.022	0.111	0.107	0.043
0.358	0.277	0.167	0.353	0.179	0.041	0.534	0.331	0.101

0.315	0.201	0.082	0.285	0.184	0.035	0.672	0.456	0.108
1.748	1.09	0.229	0.269	0.203	0.049	0.615	0.287	0.038
0.435	0.503	0.297	0.209	0.244	0.056	0.119	0.148	0.043
1.486	0.609	0.317	0.439	0.202	0.049	0.423	0.236	0.074
0.364	0.363	0.158	0.345	0.341	0.061	0.118	0.118	0.054
0.434	0.339	0.114	0.476	0.329	0.064	0.169	0.142	0.038
0.354	0.314	0.147	0.292	0.207	0.055	0.302	0.233	0.085
0.578	0.447	0.148	0.517	0.385	0.078	0.118	0.103	0.03
0.35	0.265	0.169	0.465	0.272	0.061	0.328	0.17	0.027
0.222	0.196	0.101	0.196	0.151	0.029	0.35	0.276	0.074
0.578	0.301	0.168	0.582	0.266	0.061	0.284	0.125	0.027
0.334	0.305	0.118	0.314	0.267	0.058	0.191	0.156	0.043
0.273	0.379	0.078	0.22	0.284	0.054	0.045	0.065	0.014
1.039	0.621	0.258	0.44	0.156	0.027	0.937	0.494	0.15
0.495	0.437	0.094	0.229	0.214	0.033	0.268	0.255	0.063
0.542	0.322	0.087	0.261	0.181	0.045	0.434	0.283	0.063
1.089	0.505	0.18	0.638	0.175	0.022	1.679	0.611	0.126
0.44	0.353	0.135	0.392	0.252	0.041	0.159	0.139	0.095
0.681	0.586	0.197	0.59	0.463	0.111	0.202	0.089	0.055
0.412	0.618	0.216	0.208	0.304	0.06	0.199	0.277	0.061
0.547	0.227	0.071	0.396	0.252	0.024	0.146	0.098	0.034
0.99	0.663	0.197	0.661	0.353	0.065	0.513	0.318	0.083
0.272	0.342	0.107	0.186	0.237	0.048	0.233	0.3	0.069
0.583	0.465	0.124	0.609	0.501	0.049	0.258	0.264	0.138
0.829	0.612	0.215	0.735	0.343	0.066	0.502	0.322	0.09
0.198	0.214	0.123	0.156	0.261	0.055	0.081	0.138	0.041
0.676	0.673	0.255	0.305	0.3	0.072	0.271	0.268	0.072
0.358	0.378	0.183	0.171	0.187	0.052	0.167	0.199	0.036
0.502	0.436	0.096	0.273	0.245	0.049	0.246	0.213	0.048
0.28	0.243	0.112	0.248	0.193	0.042	0.09	0.08	0.028
0.905	0.275	0.115	0.764	0.201	0.03	1.084	0.305	0.073
0.424	0.336	0.126	0.363	0.258	0.055	0.105	0.062	0.011
0.428	0.338	0.127	0.275	0.185	0.032	0.571	0.382	0.099
0.281	0.255	0.118	0.304	0.277	0.067	0.248	0.218	0.046
0.429	0.207	0.009	0.505	0.192	0.03	0.539	0.186	0.041
0.204	0.257	0.058	0.144	0.158	0.049	0.232	0.278	0.086
0.384	0.336	0.109	0.319	0.286	0.089	0.438	0.378	0.069
0.938	0.318	0.332	0.461	0.318	0.057	0.222	0.151	0.045
0.543	0.465	0.219	0.152	0.119	0.034	0.488	0.371	0.091
1.414	1.323	0.564	0.34	0.305	0.054	0.242	0.224	0.045
0.372	0.368	0.156	0.297	0.292	0.054	0.145	0.143	0.039
0.521	0.302	0.114	0.441	0.277	0.058	0.112	0.057	0.02
1.538	0.281	0.164	0.132	0.077	0.022	0.447	0.198	0.083
1.068	0.868	0.319	0.619	0.465	0.116	0.252	0.116	0.062
0.94	0.596	0.252	0.287	0.15	0.025	0.109	0.035	0.011
0.275	0.379	0.151	0.147	0.184	0.057	0.102	0.136	0.03
0.393	0.314	0.164	0.415	0.237	0.048	0.31	0.179	0.052
0.875	0.521	0.135	0.285	0.163	0.036	0.702	0.391	0.09
0.271	0.323	0.091	0.109	0.12	0.041	0.066	0.068	0.02
0.444	0.424	0.114	0.29	0.274	0.048	0.155	0.149	0.053
0.591	0.294	0.097	0.7	0.352	0.081	0.343	0.183	0.068

0.528	0.327	0.111	0.296	0.206	0.042	0.419	0.221	0.026
0.39	0.172	0.084	0.329	0.182	0.055	0.381	0.219	0.077
0.428	0.403	0.19	0.324	0.253	0.052	0.335	0.258	0.104
0.625	0.548	0.201	0.266	0.201	0.052	0.2	0.205	0.131
0.175	0.233	0.09	0.122	0.179	0.053	0.212	0.328	0.071
0.355	0.328	0.101	0.089	0.076	0.014	0.168	0.158	0.043
0.221	0.236	0.089	0.203	0.278	0.057	0.094	0.132	0.064
0.228	0.289	0.124	0.16	0.267	0.055	0.094	0.169	0.033
0.686	0.391	0.144	0.282	0.165	0.023	0.041	0.032	0.01
0.402	0.194	0.071	0.924	0.442	0.062	0.51	0.161	0.124
0.448	0.347	0.139	0.467	0.26	0.031	0.156	0.099	0.081
0.566	0.33	0.185	0.265	0.117	0.02	0.516	0.245	0.051
0.264	0.234	0.103	0.338	0.247	0.086	0.953	0.524	0.166
1.449	0.522	0.273	0.32	0.248	0.047	0.188	0.121	0.045
0.259	0.283	0.112	0.18	0.261	0.04	0.104	0.138	0.04
0.263	0.314	0.124	0.095	0.121	0.033	0.153	0.188	0.021
0.421	0.404	0.161	0.272	0.254	0.049	0.125	0.115	0.026
0.908	0.47	0.174	0.694	0.262	0.043	0.802	0.261	0.044
0.299	0.318	0.14	0.151	0.164	0.059	0.066	0.068	0.065
0.415	0.512	0.12	0.235	0.274	0.062	0.21	0.234	0.065
0.29	0.389	0.114	0.185	0.266	0.061	0.197	0.258	0.088
0.7	0.384	0.28	0.763	0.229	0.072	0.759	0.223	0.09
0.189	0.217	0.081	0.214	0.276	0.056	0.142	0.17	0.051
0.444	0.203	0.043	0.303	0.183	0.062	0.428	0.249	0.086
0.702	0.396	0.197	0.54	0.206	0.051	0.542	0.168	0.027
0.294	0.347	0.157	0.138	0.165	0.025	0.099	0.134	0.047
0.428	0.758	0.249	0.38	0.224	0.045	0.806	0.486	0.133
0.452	0.286	0.097	0.434	0.177	0.027	0.477	0.238	0.057
0.74	0.54	0.219	0.406	0.259	0.03	0.49	0.286	0.069
0.216	0.23	0.095	0.169	0.175	0.046	0.094	0.119	0.069
0.241	0.29	0.186	0.059	0.134	0.039	0.125	0.336	0.076
0.479	0.432	0.237	0.555	0.339	0.038	0.24	0.22	0.075
0.413	0.273	0.09	0.534	0.296	0.067	0.811	0.452	0.117
0.51	0.386	0.15	0.299	0.215	0.05	0.359	0.252	0.066
0.396	0.212	0.071	0.554	0.265	0.043	0.437	0.225	0.056
0.789	0.697	0.282	0.274	0.22	0.039	0.274	0.204	0.046
0.187	0.21	0.074	0.156	0.166	0.046	0.199	0.215	0.069
0.305	0.232	0.144	0.381	0.138	0.024	0.339	0.137	0.041
0.317	0.253	0.08	0.24	0.171	0.3	0.597	0.458	0.13
0.739	0.457	0.149	0.382	0.184	0.045	0.691	0.297	0.071
0.129	0.169	0.099	0.052	0.161	0.027	0.114	0.294	0.067
0.606	0.464	0.244	0.147	0.085	0.3	0.221	0.122	0.033
0.559	0.371	0.214	0.367	0.156	0.019	0.376	0.226	0.067
0.494	0.304	0.103	0.396	0.183	0.036	0.531	0.284	0.081
0.616	0.273	0.104	0.365	0.221	0.055	0.522	0.089	0.014
0.863	0.51	0.224	0.389	0.126	0.017	0.155	0.105	0.068
0.627	0.388	0.08	0.338	0.201	0.032	0.631	0.394	0.136
0.776	0.384	0.191	0.311	0.212	0.052	0.386	0.038	0.019
0.237	0.183	0.07	0.344	0.206	0.047	0.226	0.121	0.017
0.39	0.224	0.085	0.536	0.226	0.038	0.47	0.198	0.03
0.797	0.336	0.134	0.791	0.23	0.047	0.473	0.197	0.07

0.199	0.211	0.116	0.079	0.138	0.036	0.193	0.318	0.096
0.533	0.33	0.114	0.414	0.237	0.051	0.114	0.07	0.035
0.675	0.369	0.161	0.441	0.136	0.007	0.382	0.195	0.051
0.649	0.523	0.23	0.161	0.121	0.024	0.368	0.263	0.053
0.399	0.433	0.146	0.322	0.339	0.053	0.083	0.079	0.025
0.305	0.247	0.137	0.463	0.18	0.03	0.315	0.096	0.031
0.704	0.474	0.102	0.196	0.16	0.054	0.206	0.122	0.019
0.646	0.523	0.187	0.124	0.086	0.009	0.487	0.391	0.126
0.487	0.474	0.204	0.249	0.231	0.048	0.337	0.317	0.08
0.522	0.488	0.196	0.325	0.305	0.061	0.159	0.152	0.052
0.503	0.405	0.212	0.301	0.218	0.046	0.401	0.294	0.06
0.949	0.311	0.103	0.547	0.296	0.057	0.154	0.067	0.017
0.407	0.344	0.126	0.258	0.203	0.035	0.174	0.159	0.065
0.713	0.453	0.176	0.352	0.2	0.05	0.432	0.218	0.04
0.543	0.308	0.09	0.744	0.42	0.042	0.352	0.167	0.057
0.62	0.502	0.205	0.251	0.201	0.053	0.314	0.257	0.07
0.273	0.204	0.11	0.235	0.147	0.037	0.248	0.154	0.034
0.211	0.214	0.08	0.251	0.297	0.037	0.06	0.06	0.061
0.532	0.494	0.189	0.179	0.161	0.031	0.353	0.315	0.079
0.543	0.166	0.061	0.372	0.157	0.029	0.424	0.142	0.02
0.567	0.434	0.194	0.231	0.128	0.015	0.585	0.393	0.176
1.712	0.92	0.548	0.372	0.088	0.027	0.683	0.173	0.03
0.572	0.418	0.093	0.666	0.509	0.056	0.253	0.256	0.032
0.503	0.416	0.161	0.533	0.413	0.065	0.124	0.133	0.021

C.Tre (M)	C.Tre (6mm)	C.Tre (4mm)	C.HOA (M)	C.HOA (6mm)	C.HOA (4mm)	I.Sph (M)	I.Sph (6mm)	I.Sph (4mm)
0.079	0.092	0.055	0.334	0.424	0.118	0.248	0.369	0.041
0.295	0.267	0.057	0.821	0.588	0.128	0.444	0.225	0.02
0.395	0.204	0.03	0.747	0.421	0.128	0.278	0.092	0.022
0.527	0.226	0.049	1.078	0.447	0.105	0.579	0.165	0.064
0.158	0.156	0.11	0.514	0.37	0.147	0.429	0.248	0.019
0.248	0.178	0.062	0.466	0.348	0.099	0.145	0.071	0.006
0.232	0.221	0.045	0.377	0.347	0.087	0.051	0.05	0.043
0.093	0.251	0.03	0.375	0.574	0.182	0.222	0.283	0.055
0.291	0.223	0.033	0.67	0.397	0.093	0.497	0.186	0.045
0.228	0.133	0.044	0.761	0.329	0.081	0.771	0.283	0.055
0.204	0.17	0.096	0.679	0.42	0.129	0.284	0.149	0.034
0.111	0.091	0.041	0.577	0.38	0.101	0.167	0.107	0.022
0.218	0.233	0.037	0.556	0.585	0.123	0.096	0.1	0.015
0.261	0.153	0.033	0.866	0.6	0.173	0.558	0.332	0.047
0.295	0.213	0.054	0.718	0.492	0.089	0.391	0.174	0.013
0.299	0.186	0.055	0.659	0.361	0.086	0.776	0.192	0.035
0.298	0.2	0.061	0.915	0.333	0.09	0.51	0.129	0.034
0.231	0.221	0.06	0.438	0.414	0.105	0.073	0.069	0.028
0.176	0.126	0.061	0.49	0.42	0.127	0.439	0.351	0.047
0.127	0.092	0.044	0.401	0.279	0.126	0.527	0.352	0.052
0.201	0.162	0.065	0.484	0.374	0.098	0.212	0.152	0.032
0.215	0.099	0.032	1.238	0.524	0.129	0.842	0.252	0.064
0.128	0.102	0.042	0.381	0.297	0.087	0.321	0.219	0.018
0.389	0.166	0.066	0.873	0.343	0.142	0.441	0.153	0.031
0.258	0.21	0.043	0.701	0.548	0.112	0.071	0.074	0.016
0.097	0.076	0.069	0.41	0.309	0.117	0.58	0.406	0.061
0.262	0.101	0.013	0.742	0.342	0.085	0.138	0.019	0.002
0.36	0.224	0.071	0.703	0.375	0.116	0.53	0.177	0.017
0.141	0.122	0.082	0.725	0.601	0.19	0.491	0.384	0.045
0.489	0.285	0.107	0.824	0.457	0.152	0.419	0.143	0.031
0.143	0.133	0.079	0.501	0.341	0.118	0.332	0.188	0.006
0.124	0.06	0.028	0.505	0.345	0.108	0.118	0.081	0.012
0.182	0.164	0.044	0.442	0.353	0.068	0.336	0.278	0.045
0.256	0.223	0.086	0.443	0.365	0.11	0.38	0.31	0.072
0.31	0.306	0.079	0.528	0.522	0.152	0.255	0.257	0.081
0.177	0.156	0.047	0.84	0.779	0.187	0.515	0.475	0.102
0.26	0.243	0.106	0.4	0.365	0.125	0.325	0.292	0.051
0.284	0.178	0.072	0.877	0.353	0.099	0.724	0.246	0.047
0.07	0.094	0.03	0.285	0.385	0.106	0.025	0.038	0.023
0.16	0.147	0.064	0.785	0.366	0.127	0.679	0.243	0.05
0.166	0.176	0.061	0.268	0.293	0.113	0.266	0.324	0.025
0.391	0.274	0.094	0.564	0.4	0.151	0.268	0.178	0.04
0.417	0.204	0.067	1.176	0.485	0.133	0.473	0.15	0.023
0.098	0.167	0.074	0.222	0.381	0.153	0.106	0.176	0.066
0.308	0.049	0.021	1.024	0.299	0.076	0.748	0.124	0.027
0.034	0.036	0.01	0.502	0.547	0.139	0.136	0.169	0.02
0.122	0.113	0.031	0.411	0.371	0.113	0.158	0.14	0.017
0.458	0.342	0.137	0.849	0.66	0.252	0.362	0.279	0.049

0.45	0.363	0.154	0.678	0.541	0.239	0.175	0.089	0.012
0.213	0.159	0.053	0.463	0.306	0.074	0.351	0.231	0.075
0.236	0.155	0.027	0.434	0.274	0.067	0.122	0.025	0.017
0.114	0.075	0.05	0.84	0.278	0.096	0.645	0.169	0.033
0.133	0.135	0.057	0.413	0.416	0.167	0.191	0.192	0.048
0.426	0.358	0.042	0.897	0.77	0.094	0.404	0.348	0.031
0.105	0.106	0.071	0.336	0.404	0.108	0.266	0.378	0.037
0.054	0.059	0.03	0.416	0.445	0.115	0.158	0.175	0.023
0.446	0.223	0.068	0.934	0.385	0.091	0.41	0.085	0.004
0.158	0.184	0.05	0.45	0.515	0.147	0.122	0.127	0.061
0.269	0.185	0.064	0.512	0.299	0.076	0.424	0.257	0.024
0.123	0.166	0.061	0.269	0.46	0.113	0.05	0.067	0.006
0.39	0.326	0.125	0.598	0.48	0.153	0.371	0.254	0.012
0.366	0.128	0.042	1.095	0.304	0.084	1.201	0.191	0.061
1.153	0.447	0.033	2.283	1.012	0.214	0.723	0.359	0.08
0.618	0.573	0.076	1.307	1.199	0.136	0.704	0.584	0.021
0.244	0.098	0.042	0.841	0.329	0.095	0.552	0.196	0.075
0.419	0.06	0.026	1.563	0.76	0.155	0.727	0.226	0.02
0.282	0.286	0.108	1.336	0.839	0.218	0.647	0.32	0.076
0.724	0.207	0.105	1.446	0.483	0.193	0.347	0.086	0.053
0.095	0.061	0.025	0.483	0.286	0.071	0.323	0.093	0.031
0.233	0.183	0.083	0.405	0.252	0.089	0.379	0.164	0.008
0.105	0.075	0.021	0.493	0.316	0.083	0.349	0.227	0.065
0.203	0.216	0.145	0.839	0.737	0.311	0.128	0.049	0.006
0.203	0.121	0.048	0.597	0.292	0.072	0.399	0.176	0.031
0.316	0.282	0.098	0.417	0.367	0.121	0.119	0.106	0.039
0.609	0.4	0.144	0.817	0.481	0.156	0.167	0.116	0.031
0.299	0.282	0.096	0.496	0.46	0.122	0.278	0.258	0.048
0.116	0.12	0.045	0.452	0.48	0.113	0.144	0.152	0.03
0.306	0.305	0.292	0.82	0.827	0.459	0.209	0.218	0.143
0.093	0.108	0.102	0.634	0.362	0.14	0.25	0.119	0.032
0.343	0.328	0.092	0.471	0.45	0.174	0.147	0.149	0.138
0.146	0.165	0.044	0.359	0.413	0.104	0.15	0.194	0.022
0.109	0.083	0.067	0.387	0.303	0.139	0.351	0.351	0.208
0.046	0.031	0.021	0.709	0.375	0.123	0.294	0.111	0.019
0.3	0.039	0.033	1.44	0.521	0.145	0.37	0.064	0.02
0.147	0.071	0.027	1.116	0.515	0.133	0.702	0.266	0.061
0.151	0.157	0.041	0.395	0.411	0.094	0.222	0.224	0.055
0.835	0.328	0.072	1.905	0.702	0.18	0.227	0.019	0.007
0.103	0.101	0.03	0.481	0.364	0.093	0.374	0.251	0.045
0.08	0.087	0.034	0.39	0.267	0.081	0.195	0.094	0.004
0.164	0.086	0.036	0.945	0.529	0.138	0.365	0.148	0.035
0.248	0.137	0.049	0.986	0.445	0.106	0.664	0.146	0.019
0.769	0.267	0.134	1.646	0.716	0.284	0.209	0.157	0.112
0.187	0.186	0.052	0.401	0.397	0.094	0.286	0.282	0.012
0.241	0.255	0.074	0.411	0.439	0.118	0.142	0.16	0.009
0.078	0.065	0.034	0.392	0.345	0.099	0.099	0.088	0.015
0.094	0.081	0.019	0.558	0.476	0.139	0.297	0.252	0.065
0.115	0.107	0.028	0.384	0.353	0.081	0.29	0.231	0.015
0.196	0.184	0.044	0.309	0.291	0.078	0.127	0.113	0.018
0.149	0.133	0.102	0.659	0.401	0.154	0.356	0.177	0.059

0.133	0.094	0.029	0.745	0.504	0.12	0.178	0.121	0.022
0.218	0.086	0.038	0.744	0.381	0.091	0.737	0.431	0.044
0.141	0.159	0.051	0.298	0.35	0.104	0.119	0.16	0.018
0.375	0.225	0.086	0.727	0.388	0.13	0.707	0.154	0.034
0.225	0.224	0.069	0.435	0.431	0.111	0.191	0.188	0.017
0.043	0.059	0.045	0.531	0.377	0.095	0.319	0.209	0.043
0.156	0.126	0.043	0.452	0.339	0.112	0.251	0.184	0.048
0.279	0.221	0.058	0.609	0.466	0.11	0.574	0.301	0.054
0.314	0.195	0.052	0.664	0.386	0.091	0.453	0.234	0.043
0.128	0.114	0.044	0.435	0.348	0.103	0.161	0.115	0.007
0.271	0.132	0.029	0.73	0.338	0.094	0.5	0.171	0.04
0.131	0.122	0.054	0.409	0.349	0.098	0.166	0.13	0.011
0.19	0.233	0.035	0.3	0.38	0.076	0.352	0.517	0.035
0.204	0.138	0.059	1.064	0.542	0.168	0.965	0.384	0.067
0.079	0.076	0.02	0.372	0.35	0.08	0.442	0.386	0.019
0.187	0.17	0.078	0.563	0.388	0.114	0.087	0.044	0.002
0.844	0.329	0.075	2.021	0.722	0.15	0.269	0.07	0.051
0.165	0.127	0.068	0.463	0.32	0.128	0.396	0.26	0.058
0.307	0.282	0.08	0.735	0.59	0.156	0.193	0.136	0.025
0.133	0.176	0.04	0.336	0.468	0.108	0.275	0.514	0.088
0.054	0.058	0.048	0.439	0.284	0.07	0.194	0.184	0.016
0.566	0.348	0.106	1.032	0.6	0.151	0.469	0.187	0.028
0.176	0.197	0.084	0.362	0.451	0.123	0.155	0.214	0.022
0.069	0.077	0.115	0.679	0.585	0.195	0.405	0.285	0.003
0.558	0.324	0.104	1.07	0.582	0.157	0.536	0.195	0.037
0.12	0.177	0.047	0.223	0.359	0.093	0.253	0.278	0.081
0.359	0.356	0.107	0.563	0.556	0.157	0.026	0.027	0.013
0.13	0.134	0.068	0.274	0.307	0.104	0.086	0.092	0.034
0.132	0.132	0.074	0.425	0.381	0.119	0.275	0.242	0.023
0.078	0.064	0.031	0.287	0.228	0.068	0.252	0.185	0.039
0.077	0.057	0.017	1.355	0.375	0.086	1.5	0.354	0.032
0.223	0.169	0.057	0.448	0.319	0.084	0.329	0.223	0.047
0.127	0.087	0.029	0.669	0.447	0.114	0.301	0.202	0.056
0.174	0.166	0.073	0.448	0.408	0.116	0.288	0.253	0.043
0.13	0.082	0.034	0.756	0.285	0.065	0.572	0.172	0.034
0.072	0.084	0.068	0.309	0.368	0.124	0.232	0.253	0.061
0.318	0.266	0.079	0.654	0.565	0.156	0.197	0.165	0.058
0.468	0.386	0.138	0.729	0.549	0.172	0.095	0.058	0.038
0.126	0.092	0.029	0.539	0.413	0.107	0.22	0.185	0.044
0.201	0.189	0.056	0.478	0.437	0.097	0.562	0.518	0.027
0.281	0.277	0.06	0.443	0.436	0.09	0.194	0.19	0.01
0.332	0.222	0.061	0.627	0.394	0.099	0.8	0.467	0.077
0.268	0.184	0.107	0.662	0.353	0.147	0.622	0.106	0.01
0.363	0.309	0.097	0.81	0.608	0.18	0.273	0.163	0.03
0.498	0.313	0.126	0.591	0.351	0.131	0.156	0.087	0.009
0.113	0.134	0.057	0.217	0.272	0.087	0.053	0.069	0.04
0.277	0.184	0.062	0.597	0.356	0.099	0.465	0.225	0.017
0.799	0.435	0.114	1.112	0.613	0.151	0.301	0.12	0.017
0.196	0.222	0.08	0.285	0.354	0.106	0.232	0.272	0.051
0.12	0.115	0.023	0.358	0.339	0.076	0.334	0.313	0.051
0.335	0.197	0.064	0.865	0.456	0.142	0.297	0.119	0.027

0.219	0.129	0.039	0.598	0.353	0.071	0.401	0.24	0.034
0.273	0.119	0.029	0.58	0.313	0.102	0.264	0.161	0.047
0.072	0.053	0.067	0.48	0.374	0.145	0.254	0.171	0.037
0.294	0.254	0.051	0.452	0.389	0.153	0.136	0.095	0.067
0.087	0.133	0.034	0.269	0.409	0.108	0.097	0.154	0.015
0.125	0.111	0.02	0.243	0.222	0.059	0.2	0.183	0.019
0.157	0.257	0.04	0.285	0.42	0.103	0.092	0.099	0.015
0.185	0.263	0.075	0.281	0.443	0.113	0.061	0.105	0.003
0.163	0.102	0.022	0.343	0.208	0.042	0.131	0.131	0.027
1.218	0.367	0.312	1.947	0.698	0.359	0.973	0.472	0.059
0.39	0.223	0.071	0.664	0.381	0.117	0.581	0.298	0.012
0.049	0.029	0.035	0.628	0.302	0.077	0.635	0.248	0.017
0.243	0.139	0.183	1.062	0.617	0.269	0.235	0.198	0.07
0.308	0.308	0.168	0.534	0.459	0.201	0.264	1.107	0.06
0.088	0.096	0.056	0.236	0.322	0.087	0.101	0.127	0.017
0.099	0.117	0.048	0.245	0.302	0.068	0.105	0.138	0.026
0.206	0.194	0.051	0.368	0.344	0.08	0.208	0.196	0.041
0.481	0.254	0.086	1.21	0.46	0.115	0.49	0.131	0.002
0.113	0.115	0.094	0.217	0.23	0.142	0.081	0.092	0.026
0.047	0.051	0.022	0.334	0.38	0.104	0.306	0.367	0.089
0.08	0.133	0.034	0.286	0.399	0.116	0.1	0.182	0.029
0.75	0.268	0.116	1.389	0.482	0.185	0.825	0.05	0.07
0.052	0.057	0.032	0.266	0.334	0.086	0.213	0.288	0.046
0.097	0.095	0.073	0.577	0.343	0.149	0.584	0.301	0.068
0.427	0.192	0.084	0.891	0.34	0.109	0.519	0.15	0.042
0.148	0.154	0.094	0.26	0.317	0.125	0.11	0.143	0.025
0.668	0.414	0.134	1.124	0.678	0.195	0.541	0.153	0.01
0.25	0.146	0.068	0.71	0.337	0.102	0.171	0.015	0.011
0.405	0.215	0.047	0.769	0.457	0.094	0.32	0.178	0.016
0.118	0.128	0.055	0.241	0.261	0.11	0.168	0.187	0.035
0.04	0.079	0.043	0.148	0.372	0.101	0.061	0.079	0.031
0.181	0.117	0.073	0.785	0.525	0.15	0.638	0.354	0.062
0.076	0.059	0.024	0.992	0.552	0.142	0.409	0.155	0.018
0.084	0.063	0.03	0.501	0.357	0.111	0.286	0.159	0.024
0.175	0.054	0.023	0.747	0.36	0.078	0.435	0.214	0.031
0.089	0.088	0.06	0.442	0.345	0.102	0.296	0.227	0.016
0.191	0.201	0.067	0.325	0.347	0.114	0.072	0.081	0.019
0.203	0.048	0.006	0.595	0.243	0.058	0.436	0.083	0.014
0.183	0.146	0.069	0.677	0.517	0.154	0.363	0.229	0.027
0.326	0.201	0.052	0.868	0.409	0.101	0.103	0.021	0.022
0.054	0.121	0.034	0.143	0.367	0.084	0.041	0.084	0.021
0.172	0.094	0.021	0.374	0.215	0.055	0.318	0.151	0.017
0.236	0.133	0.045	0.6	0.328	0.091	0.537	0.191	0.016
0.212	0.124	0.031	0.697	0.361	0.097	0.532	0.193	0.018
0.215	0.084	0.023	0.808	0.273	0.073	0.725	0.298	0.062
0.137	0.057	0.049	0.466	0.196	0.1	0.438	0.135	0.016
0.332	0.225	0.095	0.808	0.506	0.172	0.264	0.109	0.017
0.431	0.264	0.084	0.792	0.357	0.108	0.681	0.32	0.052
0.089	0.046	0.034	0.439	0.25	0.066	0.386	0.218	0.034
0.211	0.102	0.022	0.757	0.331	0.07	0.555	0.184	0.021
0.375	0.177	0.049	1.006	0.356	0.104	0.885	0.199	0.049

0.041	0.076	0.027	0.222	0.364	0.118	0.099	0.107	0.054
0.456	0.248	0.052	0.662	0.38	0.087	0.518	0.225	0.041
0.274	0.122	0.063	0.693	0.282	0.09	0.421	0.146	0.033
0.188	0.13	0.053	0.476	0.337	0.093	0.364	0.273	0.073
0.087	0.096	0.058	0.383	0.403	0.09	0.19	0.21	0.037
0.22	0.081	0.023	0.63	0.241	0.071	0.41	0.081	0.009
0.196	0.136	0.056	0.409	0.257	0.106	0.273	0.206	0.057
0.146	0.099	0.024	0.544	0.428	0.136	0.295	0.226	0.054
0.285	0.271	0.085	0.514	0.484	0.131	0.204	0.181	0.018
0.231	0.221	0.06	0.438	0.414	0.105	0.073	0.069	0.028
0.038	0.032	0.038	0.517	0.38	0.09	0.251	0.166	0.026
0.299	0.186	0.055	0.659	0.361	0.086	0.776	0.192	0.035
0.423	0.351	0.115	0.572	0.472	0.148	0.342	0.272	0.07
0.638	0.356	0.089	0.851	0.469	0.115	0.119	0.066	0.046
0.147	0.143	0.045	0.851	0.482	0.086	0.417	0.179	0.014
0.376	0.316	0.104	0.557	0.459	0.138	0.028	0.026	0.016
0.215	0.128	0.017	0.415	0.254	0.058	0.27	0.151	0.031
0.136	0.155	0.052	0.303	0.352	0.091	0.145	0.152	0.002
0.098	0.088	0.041	0.433	0.389	0.109	0.319	0.279	0.02
0.083	0.061	0.031	0.577	0.225	0.053	0.429	0.27	0.06
0.266	0.184	0.078	0.731	0.479	0.199	0.471	0.267	0.027
0.515	0.164	0.033	0.967	0.266	0.061	0.361	0.207	0.039
0.142	0.115	0.035	0.744	0.598	0.077	0.351	0.178	0.019
0.111	0.059	0.021	0.582	0.452	0.075	0.322	0.16	0.018

I.Coma (M)	I.Coma (6mm)	I.Coma (4mm)	I.Tre (M)	I.Tre (6mm)	I.Tre (4mm)	I.HOA (M)	I.HOA (6mm)	I.HOA (4mm)
0.343	0.458	0.096	0.195	0.211	0.121	0.495	0.665	0.169
0.585	0.464	0.086	0.396	0.358	0.107	0.863	0.647	0.164
0.362	0.261	0.081	0.109	0.116	0.077	0.555	0.361	0.157
0.41	0.233	0.041	0.102	0.065	0.053	0.768	0.34	0.141
0.436	0.304	0.068	0.221	0.219	0.126	0.686	0.481	0.196
0.45	0.312	0.068	0.144	0.108	0.079	0.533	0.374	0.149
0.231	0.202	0.064	0.112	0.086	0.059	0.296	0.263	0.113
0.078	0.096	0.04	0.054	0.066	0.043	0.264	0.328	0.117
0.253	0.106	0.029	0.229	0.151	0.078	0.627	0.281	0.107
0.263	0.148	0.058	0.311	0.215	0.136	0.884	0.396	0.171
0.586	0.339	0.056	0.32	0.244	0.112	0.763	0.475	0.162
0.638	0.416	0.117	0.385	0.285	0.118	0.811	0.534	0.184
0.254	0.259	0.056	0.474	0.512	0.091	0.648	0.701	0.114
0.322	0.285	0.147	0.767	0.532	0.202	1.063	0.738	0.276
0.233	0.149	0.039	0.192	0.16	0.066	0.51	0.307	0.119
0.551	0.156	0.045	0.308	0.151	0.048	1.254	0.317	0.088
0.796	0.241	0.056	0.28	0.138	0.095	1.016	0.331	0.131
0.226	0.208	0.062	0.218	0.211	0.168	0.387	0.363	0.192
0.119	0.12	0.113	0.34	0.228	0.079	0.615	0.471	0.161
0.226	0.197	0.095	0.315	0.226	0.09	0.699	0.489	0.182
0.308	0.238	0.056	0.231	0.21	0.115	0.458	0.367	0.143
0.64	0.44	0.121	0.414	0.274	0.122	1.193	0.585	0.188
0.252	0.189	0.059	0.116	0.114	0.04	0.441	0.324	0.09
0.588	0.175	0.038	0.251	0.227	0.191	0.814	0.356	0.226
0.374	0.306	0.095	0.148	0.165	0.123	0.473	0.411	0.195
0.357	0.258	0.101	0.259	0.139	0.132	0.878	0.593	0.221
0.492	0.245	0.053	0.221	0.153	0.061	0.597	0.318	0.104
0.166	0.1	0.049	0.145	0.137	0.066	0.677	0.282	0.1
0.595	0.442	0.068	0.147	0.145	0.05	0.795	0.61	0.106
0.288	0.185	0.033	0.228	0.122	0.05	0.591	0.3	0.089
0.124	0.091	0.049	0.165	0.113	0.066	0.554	0.359	0.155
0.152	0.159	0.073	0.154	0.088	0.071	0.338	0.229	0.126
0.343	0.291	0.082	0.129	0.117	0.034	0.514	0.438	0.125
0.329	0.283	0.071	0.061	0.056	0.043	0.513	0.431	0.128
0.235	0.241	0.089	0.15	0.159	0.039	0.419	0.428	0.165
0.154	0.158	0.079	0.194	0.184	0.159	0.684	0.641	0.257
0.214	0.195	0.029	0.053	0.05	0.106	0.414	0.372	0.125
0.818	0.344	0.076	0.564	0.338	0.112	1.259	0.557	0.164
0.316	0.401	0.111	0.109	0.135	0.058	0.353	0.457	0.137
0.553	0.244	0.083	0.118	0.064	0.012	0.898	0.365	0.11
0.235	0.271	0.075	0.331	0.362	0.147	0.517	0.601	0.177
0.188	0.169	0.048	0.269	0.198	0.067	0.518	0.386	0.122
1.135	0.409	0.112	0.542	0.297	0.086	1.41	0.557	0.149
0.23	0.212	0.073	0.258	0.319	0.239	0.41	0.512	0.281
0.528	0.249	0.073	0.673	0.299	0.113	1.158	0.426	0.154
0.294	0.324	0.092	0.067	0.06	0.006	0.362	0.406	0.122
0.253	0.193	0.066	0.227	0.193	0.07	0.435	0.35	0.127
0.466	0.323	0.052	0.345	0.294	0.131	0.785	0.606	0.172

0.465	0.36	0.124	0.158	0.14	0.09	0.58	0.435	0.186
0.268	0.163	0.034	0.073	0.073	0.045	0.469	0.311	0.106
0.313	0.207	0.068	0.253	0.171	0.042	0.436	0.276	0.086
0.34	0.139	0.06	0.279	0.18	0.083	0.823	0.311	0.121
0.284	0.286	0.157	0.513	0.514	0.223	0.639	0.642	0.307
0.466	0.396	0.088	0.548	0.461	0.025	0.946	0.821	0.465
0.279	0.396	0.041	0.106	0.109	0.073	0.421	0.602	0.11
0.285	0.305	0.072	0.188	0.19	0.094	0.382	0.405	0.125
0.463	0.227	0.058	0.176	0.135	0.049	0.667	0.305	0.101
0.291	0.318	0.085	0.262	0.285	0.092	0.597	0.65	0.229
0.27	0.187	0.057	0.462	0.327	0.166	0.799	0.548	0.227
0.221	0.252	0.097	0.081	0.099	0.033	0.289	0.325	0.16
0.43	0.322	0.07	0.276	0.202	0.063	0.641	0.47	0.108
0.41	0.133	0.027	0.617	0.154	0.077	1.559	0.298	0.11
1.34	0.736	0.138	0.983	0.547	0.095	1.935	1.071	0.219
0.775	0.662	0.063	0.704	0.648	0.065	1.401	1.226	0.115
0.3	0.109	0.051	0.354	0.269	0.161	0.761	0.373	0.192
0.934	0.33	0.107	0.201	0.125	0.013	1.225	0.427	0.119
0.765	0.398	0.121	0.436	0.367	0.109	1.23	0.667	0.2
0.707	0.117	0.039	0.522	0.275	0.101	1.006	0.332	0.155
0.105	0.073	0.025	0.139	0.119	0.071	0.426	0.212	0.099
0.388	0.233	0.045	0.259	0.206	0.111	0.621	0.367	0.124
0.425	0.304	0.092	0.278	0.197	0.108	1.332	1.067	0.619
0.253	0.175	0.071	0.408	0.357	0.151	0.645	0.527	0.252
0.428	0.242	0.074	0.263	0.178	0.054	0.668	0.363	0.116
0.176	0.147	0.057	0.152	0.127	0.053	0.304	0.258	0.102
0.363	0.191	0.033	0.465	0.284	0.081	0.635	0.372	0.106
0.293	0.256	0.043	0.261	0.239	0.047	0.526	0.474	0.106
0.265	0.281	0.052	0.232	0.242	0.12	0.423	0.44	0.153
0.666	0.669	0.323	0.502	0.504	0.364	0.969	0.974	0.618
0.406	0.254	0.073	0.371	0.24	0.111	0.612	0.374	0.142
0.344	0.316	0.116	0.498	0.474	0.092	0.674	0.638	0.174
0.302	0.346	0.088	0.143	0.164	0.054	0.385	0.452	0.117
0.258	0.208	0.096	0.601	0.473	0.211	1.112	0.979	0.488
0.401	0.21	0.038	0.203	0.121	0.055	0.571	0.289	0.077
1.113	0.46	0.158	0.567	0.304	0.126	1.351	0.577	0.22
0.489	0.228	0.061	0.095	0.036	0.031	0.872	0.365	0.107
0.11	0.112	0.046	0.111	0.121	0.064	0.361	0.373	0.13
1.118	0.383	0.084	0.32	0.112	0.078	1.247	0.41	0.128
0.261	0.216	0.1	0.19	0.111	0.067	0.622	0.453	0.165
0.26	0.139	0.04	0.103	0.038	0.032	0.383	0.22	0.095
0.695	0.408	0.083	0.322	0.213	0.065	0.892	0.506	0.135
0.703	0.276	0.053	0.597	0.629	0.357	1.254	0.74	0.39
0.618	0.308	0.177	0.621	0.465	0.191	1.136	0.764	0.434
0.312	0.308	0.05	0.183	0.182	0.055	0.484	0.477	0.097
0.219	0.229	0.083	0.435	0.437	0.282	0.543	0.558	0.322
0.165	0.129	0.048	0.253	0.232	0.091	0.402	0.339	0.119
0.355	0.313	0.108	0.149	0.14	0.078	0.521	0.457	0.165
0.219	0.204	0.074	0.34	0.327	0.258	0.533	0.49	0.312
0.096	0.078	0.054	0.084	0.081	0.032	0.23	0.204	0.081
0.323	0.182	0.052	0.196	0.182	0.164	0.534	0.321	0.187

0.448	0.306	0.058	0.19	0.14	0.073	0.537	0.368	0.104
0.484	0.252	0.061	0.54	0.32	0.101	1.41	0.994	0.218
0.2	0.235	0.069	0.293	0.307	0.269	0.421	0.514	0.296
0.794	0.333	0.12	1.331	0.688	0.325	1.964	0.866	0.415
0.12	0.119	0.031	0.396	0.394	0.178	0.473	0.471	0.193
0.466	0.367	0.099	0.232	0.197	0.04	0.622	0.478	0.125
0.095	0.048	0.027	0.059	0.061	0.055	0.361	0.281	0.123
0.268	0.169	0.06	0.229	0.199	0.088	0.738	0.444	0.124
0.362	0.172	0.059	0.275	0.251	0.138	0.726	0.444	0.2
0.373	0.286	0.06	0.185	0.164	0.107	0.49	0.394	0.147
0.23	0.182	0.075	0.414	0.205	0.102	0.847	0.424	0.213
0.229	0.195	0.062	0.058	0.056	0.044	0.337	0.288	0.11
0.156	0.227	0.043	0.046	0.065	0.02	0.397	0.58	0.08
0.656	0.314	0.102	0.482	0.358	0.183	1.293	0.633	0.238
0.333	0.28	0.063	0.127	0.128	0.038	0.587	0.512	0.082
0.341	0.215	0.052	0.296	0.184	0.066	0.476	0.299	0.098
1.093	0.458	0.09	0.58	0.102	0.054	1.407	0.503	0.159
0.289	0.206	0.057	0.045	0.056	0.034	0.532	0.375	0.124
0.283	0.243	0.125	0.081	0.081	0.04	0.416	0.338	0.141
0.287	0.703	0.065	0.32	0.725	0.164	0.67	1.362	0.215
0.248	0.179	0.025	0.357	0.111	0.051	0.553	0.319	0.095
0.277	0.172	0.055	0.109	0.1	0.048	0.587	0.292	0.094
0.163	0.206	0.063	0.124	0.162	0.032	0.286	0.384	0.085
0.143	0.126	0.097	0.128	0.151	0.177	0.478	0.37	0.226
0.161	0.091	0.019	0.173	0.138	0.08	0.615	0.279	0.108
0.167	0.17	0.109	0.051	0.058	0.046	0.324	0.351	0.157
0.331	0.321	0.056	0.454	0.455	0.157	0.703	0.7	0.232
0.217	0.262	0.049	0.283	0.285	0.206	0.423	0.458	0.219
0.098	0.086	0.038	0.214	0.174	0.022	0.445	0.38	0.096
0.065	0.054	0.026	0.168	0.153	0.075	0.333	0.266	0.102
1.303	0.403	0.065	0.24	0.127	0.087	2.011	0.559	0.123
0.2	0.125	0.033	0.362	0.248	0.049	0.566	0.387	0.107
0.247	0.102	0.025	0.228	0.16	0.027	0.514	0.335	0.118
0.248	0.212	0.029	0.058	0.063	0.034	0.447	0.387	0.08
0.597	0.223	0.057	0.195	0.133	0.056	0.865	0.318	0.096
0.16	0.184	0.061	0.078	0.101	0.06	0.328	0.38	0.116
0.361	0.301	0.024	0.274	0.216	0.052	0.583	0.479	0.105
0.177	0.142	0.033	0.325	0.295	0.17	0.446	0.415	0.248
0.279	0.221	0.064	0.457	0.37	0.133	0.645	0.536	0.223
0.815	0.75	0.075	0.671	0.603	0.362	1.719	1.574	0.521
0.336	0.331	0.099	0.258	0.258	0.121	0.504	0.498	0.174
0.16	0.149	0.075	0.188	0.218	0.08	0.883	0.553	0.15
1.116	0.2	0.039	0.342	0.086	0.025	1.76	0.311	0.125
0.446	0.328	0.116	0.461	0.365	0.147	0.754	0.561	0.222
0.372	0.265	0.1	0.289	0.24	0.135	0.579	0.4	0.181
0.137	0.171	0.063	0.073	0.089	0.069	0.259	0.341	0.157
0.362	0.237	0.073	0.11	0.112	0.077	0.622	0.366	0.138
0.192	0.09	0.032	0.165	0.056	0.005	0.452	0.214	0.075
0.164	0.195	0.04	0.204	0.25	0.036	0.425	0.51	0.102
0.288	0.277	0.08	0.278	0.27	0.094	0.563	0.536	0.148
0.424	0.202	0.078	0.128	0.143	0.067	0.596	0.3	0.135

0.661	0.388	0.081	0.296	0.15	0.075	0.86	0.502	0.125
0.501	0.321	0.135	0.1	0.019	0.027	0.585	0.368	0.155
0.359	0.331	0.18	0.281	0.265	0.104	0.542	0.471	0.216
0.305	0.305	0.167	0.165	0.149	0.083	0.663	0.599	0.281
0.161	0.195	0.073	0.037	0.065	0.032	0.221	0.29	0.104
0.135	0.133	0.08	0.08	0.08	0.042	0.325	0.303	0.107
0.061	0.07	0.031	0.214	0.23	0.067	0.263	0.283	0.097
0.122	0.134	0.068	0.081	0.109	0.035	0.228	0.31	0.109
0.32	0.234	0.064	0.58	0.376	0.147	0.775	0.488	0.17
0.569	0.164	0.161	1.065	0.342	0.328	1.903	0.692	0.385
0.339	0.226	0.086	0.182	0.082	0.072	0.737	0.402	0.13
0.506	0.217	0.034	0.335	0.294	0.205	0.895	0.448	0.212
0.999	0.591	0.19	0.251	0.275	0.243	1.081	0.7	0.322
0.208	0.853	0.062	0.093	0.268	0.056	0.445	1.647	0.153
0.211	0.233	0.089	0.129	0.145	0.039	0.287	0.324	0.112
0.136	0.134	0.057	0.252	0.263	0.076	0.344	0.374	0.117
0.201	0.19	0.058	0.115	0.113	0.074	0.348	0.329	0.124
0.773	0.269	0.053	0.306	0.162	0.086	1.005	0.355	0.105
0.263	0.297	0.065	0.091	0.092	0.088	0.32	0.362	0.137
0.195	0.23	0.056	0.284	0.334	0.074	0.491	0.594	0.156
0.164	0.28	0.035	0.071	0.119	0.029	0.255	0.431	0.075
0.463	0.196	0.133	0.718	0.382	0.25	1.244	0.538	0.335
0.142	0.155	0.071	0.09	0.089	0.061	0.323	0.395	0.126
0.355	0.166	0.085	0.122	0.078	0.059	0.707	0.373	0.157
0.601	0.234	0.077	0.246	0.2	0.121	0.85	0.36	0.163
0.184	0.268	0.04	0.134	0.14	0.064	0.309	0.409	0.144
0.399	0.06	0.035	0.305	0.204	0.095	0.955	0.275	0.108
0.488	0.264	0.077	0.065	0.057	0.033	0.569	0.278	0.098
0.276	0.25	0.122	0.098	0.097	0.094	0.476	0.355	0.181
0.159	0.178	0.093	0.221	0.232	0.103	0.342	0.377	0.155
0.149	0.22	0.071	0.141	0.166	0.11	0.271	0.356	0.188
0.269	0.181	0.138	0.367	0.213	0.084	1.087	0.684	0.296
0.562	0.309	0.073	0.099	0.041	0.01	0.724	0.359	0.086
0.508	0.354	0.104	0.233	0.219	0.141	0.637	0.455	0.191
0.4	0.171	0.035	0.147	0.062	0.063	0.647	0.297	0.095
0.174	0.144	0.041	0.648	0.563	0.207	0.773	0.661	0.219
0.129	0.14	0.059	0.156	0.185	0.064	0.243	0.28	0.099
0.412	0.236	0.067	0.212	0.069	0.075	0.707	0.318	0.152
0.403	0.319	0.099	0.051	0.025	0.03	0.569	0.416	0.116
0.56	0.286	0.093	0.287	0.172	0.084	0.664	0.344	0.137
0.121	0.275	0.065	0.071	0.119	0.062	0.163	0.347	0.106
0.284	0.16	0.05	0.373	0.285	0.156	0.619	0.439	0.232
0.441	0.307	0.126	0.652	0.452	0.239	0.968	0.586	0.273
0.368	0.217	0.099	0.135	0.129	0.064	0.704	0.35	0.131
0.441	0.218	0.064	0.126	0.05	0.02	0.94	0.411	0.122
0.54	0.18	0.033	0.548	0.44	0.238	0.935	0.511	0.251
0.319	0.221	0.119	0.184	0.109	0.041	0.508	0.292	0.131
0.321	0.21	0.092	0.128	0.095	0.089	0.787	0.415	0.158
0.342	0.246	0.067	0.104	0.07	0.023	0.54	0.347	0.089
0.642	0.335	0.09	0.317	0.144	0.058	0.92	0.421	0.121
0.655	0.269	0.082	0.441	0.149	0.076	1.202	0.383	0.14

0.106	0.109	0.099	0.126	0.134	0.058	0.209	0.22	0.135
0.307	0.15	0.041	0.201	0.097	0.037	0.666	0.31	0.094
0.317	0.21	0.072	0.137	0.099	0.041	0.58	0.314	0.139
0.233	0.173	0.048	0.364	0.322	0.164	0.596	0.476	0.208
0.147	0.159	0.095	0.186	0.182	0.104	0.332	0.351	0.16
0.341	0.166	0.059	0.189	0.123	0.094	0.632	0.259	0.115
0.499	0.311	0.043	0.336	0.225	0.066	0.764	0.478	0.126
0.242	0.181	0.052	0.216	0.141	0.042	0.474	0.349	0.113
0.227	0.218	0.047	0.153	0.158	0.102	0.359	0.339	0.122
0.226	0.208	0.062	0.218	0.211	0.168	0.387	0.363	0.192
0.248	0.138	0.01	0.224	0.178	0.094	0.555	0.41	0.215
0.551	0.156	0.045	0.308	0.151	0.048	1.254	0.317	0.088
0.398	0.364	0.094	0.621	0.505	0.191	0.852	0.719	0.243
0.309	0.093	0.022	0.308	0.167	0.053	0.517	0.296	0.151
0.354	0.161	0.049	0.216	0.172	0.054	0.62	0.318	0.099
0.42	0.266	0.034	0.176	0.145	0.071	0.495	0.343	0.104
0.382	0.249	0.047	0.345	0.258	0.115	0.611	0.407	0.139
0.106	0.107	0.072	0.151	0.151	0.103	0.258	0.264	0.131
0.447	0.408	0.11	0.376	0.357	0.131	0.689	0.631	0.192
0.094	0.128	0.043	0.119	0.05	0.027	0.606	0.314	0.085
0.523	0.401	0.22	0.191	0.123	0.079	0.805	0.566	0.276
0.669	0.24	0.077	0.888	0.675	0.459	1.51	0.795	0.523
0.214	0.209	0.046	0.247	0.064	0.025	0.528	0.309	0.079
0.157	0.093	0.058	0.284	0.254	0.1	0.48	0.331	0.134

Q	e	SDP	SRI	SAI	MTF(6mm)
-0.05	0.23	1.24	0.71	0.41	0.155
-0.12	0.35	1.22	0.57	0.46	0.151
-0.09	0.3	1.01	0.45	0.35	0.152
-0.1	0.32	1.02	0.33	0.33	0.151
-0.06	0.24	1.24	0.78	0.32	0.151
-0.21	0.46	1.41	0.64	0.29	0.152
-0.24	0.49	1.03	0.54	0.35	0.154
-0.08	0.28	1.17	0.57	0.56	0.153
-0.19	0.43	1.18	0.41	0.28	0.151
-0.2	0.44	1.21	0.5	0.24	0.155
-0.20	0.45	1.71	0.44	0.34	0.150
-0.24	0.49	1.02	0.53	0.36	0.150
-0.21	0.45	1.28	0.55	0.36	0.156
-0.03	0.18	0.93	0.43	0.48	0.158
0.06	1.83	1.18	0.51	0.25	0.152
-0.1	0.32	0.96	0.23	0.28	0.156
-0.26	0.51	1	0.17	0.29	0.152
-0.16	0.41	1.01	0.49	0.32	0.175
0.06	-0.24	1.21	0.69	0.36	0.197
-0.18	0.42	1.17	0.68	0.41	0.182
-0.13	0.36	1.44	0.37	0.33	0.151
0.03	-0.18	0.64	0.01	0.31	0.173
-0.29	0.54	1.36	0.59	0.28	0.152
-0.25	0.5	1.06	0.45	0.4	0.152
-0.69	0.83	1.92	0.66	0.44	0.158
-0.17	0.41	1	0.67	0.4	0.17
-0.08	0.29	1.02	0.35	0.32	0.152
-0.12	0.34	0.98	0.52	0.29	0.163
-0.04	0.19	1.17	0.6	0.52	0.223
-0.33	0.57	1.04	0.33	0.33	0.15
-0.37	0.61	1.14	0.58	0.27	0.155
-0.17	0.41	0.98	0.43	0.4	0.152
-0.05	0.22	1.02	0.15	0.27	0.156
-0.23	0.48	0.72	0.32	0.29	0.16
-0.05	0.21	0.97	0.56	0.6	0.152
0.4	-0.63	1.14	0.72	0.4	0.153
-0.25	0.5	0.89	0.35	0.32	0.153
-0.16	0.4	0.93	0.25	0.29	0.164
-0.43	0.65	1.75	0.47	0.44	0.153
-0.30	0.55	1.44	0.45	0.39	0.156
-0.25	0.5	1.12	0.48	0.37	0.172
-0.13	0.36	0.98	0.62	0.31	0.162
-0.09	0.29	0.89	0.3	0.46	0.151
-0.24	0.49	1.21	0.68	0.67	0.153
-0.21	0.46	1.11	0.53	0.3	0.151
-0.11	0.33	1.13	0.54	0.53	0.161
-0.23	0.48	1.46	0.66	0.37	0.152
0.07	-0.26	1.13	0.82	0.69	0.165

-0.24	0.49	1.41	0.69	0.51	0.158
-0.13	0.36	1.07	0.24	0.25	0.156
-0.32	0.56	1.28	0.36	0.29	0.151
-0.35	0.59	1.15	0.4	0.33	0.157
-0.21	0.46	0.87	0.37	0.4	0.154
-0.27	0.52	0.97	0.68	0.46	0.171
-0.21	0.45	1.41	0.76	0.3	0.151
-0.02	0.13	1.57	0.23	0.42	0.187
-0.18	0.42	0.98	0.3	0.25	0.151
-0.32	0.56	1.02	0.55	0.4	0.16
-0.33	0.57	1.13	0.26	0.29	0.182
-0.46	0.68	1.67	0.51	0.43	0.152
-0.16	0.4	1.33	0.34	0.32	0.159
-0.21	0.46	1.18	0.2	0.23	0.17
-0.23	0.48	1.06	0.54	0.37	0.152
0	0.01	1.22	0.43	0.48	0.167
-0.05	0.23	0.87	0.11	0.31	0.15
0.27	-0.52	0.86	0.13	0.47	0.155
0.37	-0.61	1.15	0.79	0.36	0.150
-0.06	0.25	1.43	0.7	0.44	0.152
-0.17	0.41	1	0.26	0.23	0.152
-0.33	0.58	1.47	0.37	0.27	0.164
-0.21	0.46	1.41	0.5	0.38	0.178
0.23	-0.48	1.07	0.64	0.76	0.23
-0.36	0.6	1.11	0.33	0.29	0.162
-0.32	0.57	1.11	0.44	0.27	0.155
-0.79	0.89	1.71	0.75	0.33	0.158
-0.25	0.5	1.11	0.47	0.33	0.159
-0.19	0.44	1.49	0.78	0.29	0.153
-0.4	0.63	1.67	0.71	0.73	0.2
-0.17	0.42	1.16	0.49	0.37	0.163
-0.36	0.6	1.03	0.81	0.46	0.173
-0.27	0.51	1.2	0.13	0.38	0.176
-0.31	0.56	1.12	0.66	0.45	0.189
-0.26	0.51	1.21	0.36	0.46	0.159
-0.31	0.55	1.12	0.41	0.56	0.16
0	-0.03	0.83	0.2	0.33	0.292
-0.05	0.23	0.81	0.25	0.24	0.167
-0.01	0.1	1.3	0.51	0.5	0.151
-0.29	0.54	1.16	0.6	0.29	0.152
-0.26	0.51	0.97	0.43	0.35	0.155
-0.14	0.37	1.52	0.66	0.45	0.151
-0.05	0.23	0.77	0.19	0.36	0.153
-0.42	0.65	1.67	1.22	0.42	0.178
-0.17	0.41	0.87	0.27	0.31	0.161
-0.12	0.35	1.25	0.54	0.32	0.183
-0.13	0.36	1.22	0.38	0.26	0.152
-0.42	0.65	1.5	0.54	0.52	0.167
-0.21	0.45	0.99	0.27	0.32	0.224
-0.30	0.54	1.01	0.34	0.32	0.156
-0.25	0.5	1.31	0.4	0.45	0.152

-0.28	0.53	1.12	0.59	0.49	0.155
-0.14	0.38	1.13	0.43	0.34	0.176
-0.21	0.45	1.41	0.67	0.32	0.15
-0.24	0.49	1.3	0.53	0.37	0.151
-0.05	0.22	0.66	0.26	0.3	0.151
-0.05	0.21	0.87	0.18	0.27	0.151
-0.08	0.28	1.11	0.17	0.37	0.151
0.02	-0.15	0.83	0.22	0.27	0.157
-0.17	0.41	1	0.06	0.28	0.173
-0.22	0.47	1.22	0.31	0.37	0.152
-0.2	0.44	0.99	0.47	0.3	0.163
-0.17	0.41	0.98	0.09	0.29	0.155
-0.19	0.44	1.27	0.29	0.29	0.187
-0.29	0.54	0.92	0.36	0.55	0.328
-0.30	0.54	1.23	0.23	0.33	0.207
-0.28	0.53	0.92	0.54	0.36	0.187
-0.85	0.92	1.17	0.61	0.48	0.158
-0.02	0.15	1	0.29	0.41	0.155
0.08	-0.29	1.22	0.67	0.36	0.16
-0.3	0.54	1.01	0.52	0.31	0.15
-0.12	0.34	1.01	0.28	0.25	0.153
0	0.05	1.12	0.23	0.38	0.16
-0.29	0.54	1.36	0.08	0.39	0.188
0.27	-0.52	0.76	0.52	0.52	0.154
-0.01	0.11	1.08	0.41	0.38	0.155
-0.17	0.42	1.38	0.72	0.32	0.137
-0.14	0.38	1.1	0.75	0.33	0.153
-0.31	0.56	2.07	0.6	0.47	0.165
-0.22	0.47	1.51	0.76	0.34	0.159
-0.25	0.5	1.13	0.15	0.26	0.153
-0.15	0.39	1.02	0.36	0.34	0.312
-0.22	0.47	1.2	0.7	0.24	0.168
-0.28	0.53	1.15	0.51	0.46	0.171
-0.13	0.37	0.97	0.31	0.31	0.182
-0.24	0.49	0.94	0	0.3	0.219
-0.28	0.53	1.02	0.36	0.4	0.16
-0.28	0.53	1.08	0.81	0.35	0.17
-0.14	0.38	1.24	0.75	0.31	0.151
-0.26	0.51	1.12	0.46	0.42	0.164
-0.38	0.62	0.79	0.39	0.31	0.163
-0.14	0.38	0.73	0.31	0.32	0.167
-0.12	0.35	1.15	0.15	0.24	0.17
-0.46	0.68	1.33	0.72	0.38	0.16
0.09	-0.3	1.27	0.66	0.34	0.157
-0.30	0.55	1.14	0.06	0.25	0.151
-0.29	0.54	0.88	0.16	0.27	0.165
-0.07	0.26	0.99	0.17	0.32	0.167
0.02	-0.13	1.24	0.24	0.38	0.19
-0.43	0.66	1.49	0.43	0.26	0.152
-0.06	0.24	0.73	0	0.32	0.191
-0.05	0.23	1.1	0.55	0.34	0.162

-0.20	0.45	0.96	0.35	0.31	0.163
-0.19	0.44	1.1	0.25	0.38	0.151
-0.25	0.5	1.51	0.98	0.44	0.165
-0.28	0.53	1.06	0.76	0.45	0.163
-0.27	0.52	0.87	0.32	0.39	0.171
-0.44	0.66	1.27	0.49	0.3	0.173
-0.05	0.23	0.7	0.31	0.31	0.163
-0.17	0.42	0.88	0.28	0.28	0.154
-0.27	0.52	1.07	0.26	0.25	0.154
0.04	-0.2	1.13	0.78	0.53	0.162
-0.13	0.36	1	0.29	0.31	0.185
-0.37	0.61	1.34	0.47	0.3	0.185
-0.04	0.2	1.23	0.95	0.51	0.155
-0.09	0.3	1.12	0.69	0.29	0.179
-0.11	0.33	1.11	0.4	0.29	0.151
-0.26	0.51	1.24	0.42	0.31	0.154
-0.12	0.35	0.75	0	0.28	0.174
-0.05	0.21	0.69	0.31	0.41	0.225
-0.33	0.58	1.34	0.68	0.36	0.172
0.06	-0.25	0.82	0.34	0.39	0.214
-0.06	0.24	1.09	0.4	0.44	0.147
-0.23	0.48	1.4	0.48	0.41	0.181
-0.16	0.4	1.1	0.39	0.31	0.156
-0.19	0.43	0.88	0.51	0.41	0.192
-0.21	0.46	0.87	0.13	0.31	0.204
-0.37	0.61	0.95	0.52	0.28	0.168
-0.06	0.24	1.16	0.33	0.45	0.276
-0.18	0.43	1.04	0.61	0.37	0.159
-0.07	0.27	1.26	0.62	0.38	0.152
-0.29	0.54	1.04	0.56	0.33	0.153
-0.33	0.57	1.34	0.58	0.41	0.188
-0.26	0.51	1.04	0.69	0.57	0.2
0.04	-0.21	1.32	0.54	0.5	0.155
-0.3	0.55	1.35	0.93	0.39	0.165
-0.18	0.43	0.82	0.05	0.34	0.159
-0.29	0.53	1.57	0.69	0.35	0.166
-0.31	0.55	0.99	0.37	0.35	0.157
-0.39	0.63	1	0.53	0.34	0.16
-0.14	0.38	1.27	0.56	0.47	0.178
-0.18	0.42	0.94	0.38	0.37	0.155
-0.24	0.49	1.28	0.38	0.37	0.168
-0.48	0.69	1.32	0.48	0.27	0.159
-0.27	0.52	1.23	0.54	0.35	0.159
-0.26	0.51	1.21	0.54	0.38	0.152
-0.23	0.48	1.24	0.37	0.27	0.172
-0.33	0.58	1.08	0.41	0.36	0.164
-0.29	0.54	0.97	0.2	0.48	0.156
-0.23	0.48	1.27	0.41	0.27	0.175
-0.22	0.47	0.84	0.13	0.29	0.167
-0.22	0.47	1.07	0.46	0.29	0.161
-0.22	0.47	1.14	0.58	0.35	0.175

-0.4	0.63	1.58	0.62	0.44	0.14
-0.17	0.41	1.24	0.13	0.27	0.184
-0.39	0.62	1.43	0.74	0.39	0.151
-0.34	0.58	1.4	0.66	0.34	0.154
-0.05	0.22	1.01	0.48	0.32	0.162
-0.27	0.52	1.11	0.49	0.3	0.157
-0.36	0.6	0.99	0.59	0.29	0.154
-0.29	0.54	1.29	0.65	0.47	0.155
-0.15	0.39	1.11	0.27	0.38	0.184
-0.16	0.41	1.01	0.49	0.32	0.175
-0.28	0.53	1.09	0.23	0.3	0.158
-0.1	0.32	0.96	0.23	0.28	0.156
-0.15	0.39	1.25	0.5	0.36	0.151
-0.41	0.64	1.34	0.29	0.31	0.153
-0.15	0.38	1.05	0.27	0.34	0.158
-0.35	0.59	1.14	0.29	0.34	0.157
-0.42	0.65	1.6	0.49	0.28	0.154
-0.12	0.34	1	0.41	0.33	0.162
-0.19	0.44	1.01	0.39	0.4	0.184
-0.31	0.56	1.36	0.42	0.28	0.172
-0.01	0.09	1.56	0.87	1.05	0.198
-0.48	0.69	1.38	0.73	0.36	0.187
-0.15	0.39	0.93	0.32	0.34	0.267
-0.33	0.57	1.08	0.39	0.32	0.252
